# Transposable element–mediated evolutionary expansion of Sox2- and Brn2-binding regulatory modules for mammalian neural-cell differentiation

**DOI:** 10.1186/s13059-026-04050-w

**Published:** 2026-04-09

**Authors:** Hidenori Nishihara, Atsushi Komiya

**Affiliations:** Department of Advanced Bioscience, Faculty of Agriculture, Kindai University, 3327-204 Nakamachi, Nara, 631-8505 Japan

**Keywords:** Transposable elements, Retrotransposons, Endogenous retroviruses, Co-option, Exaptation, *Cis*-regulatory elements, Neural progenitor cells, Transcription factor

## Abstract

**Background:**

In mammalian genomes, at least several thousand copies of transposable elements (TEs) may function as enhancers or promoters that regulate gene expression, cellular processes, and development. However, it is still largely unknown how many TEs have been co-opted into regulatory processes and under which cellular situations they are functional. In particular, few studies have addressed how TE functions change during cell differentiation.

**Results:**

We analyze human TEs bound by the transcription factor Sox2 and by the neuronal transcription factor Brn2 during differentiation of embryonic stem cells into neural progenitor cells (NPC). We identify more than 20,000 copies of Sox2- or Brn2-binding TEs, including ancient SINEs/LINEs and simian-specific endogenous retroviruses, which represents two-wave evolutionary acquisition. Our results suggest that retrotransposition of the endogenous retroviruses including MER51 and MER49 has expanded the genomic prevalence of the simian-specific binding sites for Sox2 and Brn2, respectively. Epigenetics profiling suggests that approximately half of the Sox2- or Brn2-binding TEs function as potential *cis*-regulatory sequences, with a subset exhibiting clear functional transitions associated with Sox2 binding and release dynamics during neural cell differentiation. The nearest genes of NPC-specific Sox2 binding TEs are upregulated and enrich for neurogenesis-related gene ontology terms.

**Conclusions:**

The accumulation of TE-derived *cis*-regulatory elements during mammalian evolution may have contributed to the diversification and refinement of gene regulatory dynamics underlying neuronal development.

**Supplementary Information:**

The online version contains supplementary material available at 10.1186/s13059-026-04050-w.

## Background

Transposable elements (TEs) make up 30–50% of mammalian genomes, and the majority of TEs are retrotransposons that propagate via RNA intermediates; these include SINEs (short interspersed nuclear elements), LINEs (long interspersed nuclear elements), and LTR (long terminal repeat) retrotransposons including endogenous retroviruses (ERVs) [1, 2]. Although TEs have traditionally been regarded as harmful mutagens or selfish DNA [3, 4], accumulating evidence indicates that certain TEs have become co-opted into the cellular machinery, serving as a part of coding genes, gene regulatory elements, or controlling epigenetic modifications [5–11]. The LTR sequence of ERVs inherently possesses promoter or enhancer activities for their own transcription, and some have been evolutionarily co-opted serving as *cis*-regulatory elements controlling nearby gene expression [12–14]. For other classes of TEs, many TE-derived enhancers and promoters have been reported, suggesting that a subset of TEs has acquired regulatory functions during evolution and contributed to the rewiring of gene expression networks involved in cell regulation and development in mammals [13–17]. Some of the regulated genes are involved in the formation of unique mammalian tissues/organs, such as the neocortex [12, 16, 18–23]. Therefore, TEs are currently considered an important source of new *cis*-regulatory sequences that could contribute to the evolution of gene regulation leading to morphological innovation in mammals [5, 24, 25]. Notably, some TEs contributed to the spread of transcription factor–binding sites throughout the genome during evolution, which led to the establishment of an abundance of *cis*-regulatory sequences in modern genomes [13, 14, 23, 26–32].

Because *cis*-regulatory elements are responsible for determining the tissue specificity, timing, and rate of gene expression [33], a subset of TE sequences that serves as *cis*-regulatory elements are expected to be involved in the regulation of various cellular processes at different developmental stages. Indeed, a part of young TEs such as HERVK, HERVH, and SVA have *cis*-regulatory roles in embryonic stem cells (ESC) and neurons [34, 35]. Nevertheless, regulatory sequences derived from TEs have been examined still in a limited number of cell types under limited conditions. Although an abundance of evidence suggests that at least thousands of mammalian TEs have enhancer functions [13, 31, 36], it remains unclear how widespread TE-derived enhancers are among the ~ 4.5 million TE copies in the human genome. In particular, how the enhancer functions of TEs change during neural cell differentiation is largely unknown.

In mammals, especially humans, there has been a remarkable evolution of neuronal networks, as exemplified by the mammalian neocortex [37–42]. One of the most important transcription factors in neurogenesis is Sox2 [43]. Sox2 is responsible for not only maintaining cell pluripotency but also binding to a number of enhancers in concert with several other transcription factors to control gene expression during the differentiation of ESC into neural progenitor cells (NPC) [37, 44, 45]. Brn2 (POU3F2) is also an essential transcription factor acting in neocortical progenitors in mammals [37, 46]. Lack of Brn2 causes a lethal phenotype with abnormal telencephalon development in monkeys, suggesting its crucial role in neurogenesis and neocortical development in primates [47]. In contrast, loss of Brn2 does not lead to neocortical defects in mice due to compensatory functions provided by Brn1 [48, 49]. The differential importance of Brn2 between primates and mice may have arisen from the primate-specific expansion of Brn2-binding regulatory elements [47].

However, the extent to which TEs have been co-opted into these transcriptional programs remains unresolved. To address this gap, we analyzed public ChIP-seq (chromatin immunoprecipitation-sequencing) data for Sox2 during the differentiation of human ESC into NPC and those for Brn2 in NPC, in order to clarify how TEs contributed to the *cis*-regulatory networks underlying neuronal differentiation and their evolutionary expansion.

## Results and discussion

### Identification of Sox2-binding sites derived from TEs in the genomes of ESC and NPC

**Fig. 1:**
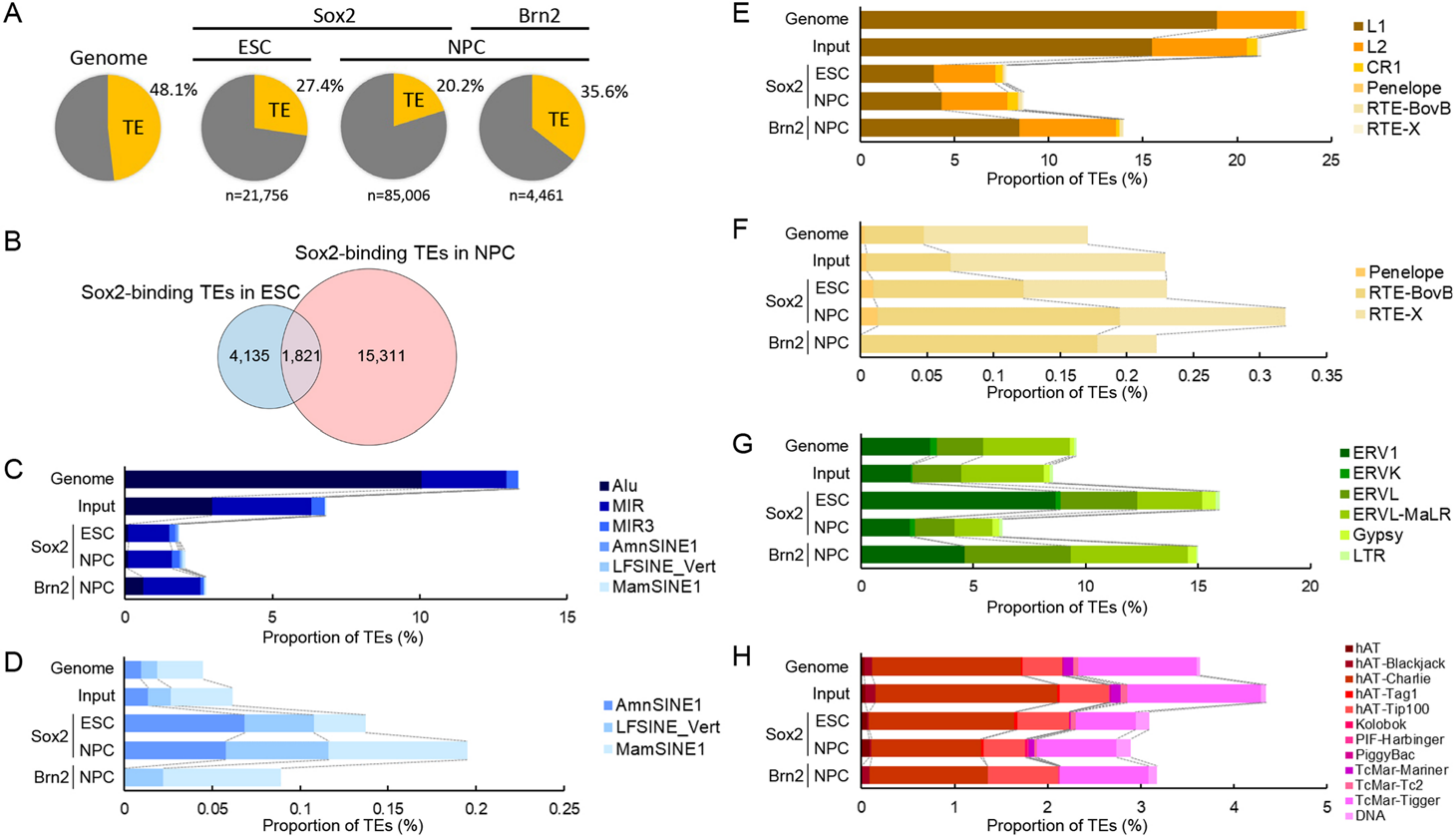
Proportions of TE types among Sox2- and Brn2-binding sites in the human genome. **A** Proportions of TEs among Sox2- and Brn2-binding sites (ChIP-seq peak summits) in ESC and NPC. **B** Numbers of Sox2-binding sites within TEs in ESC, NPC, and both cell types. **B** Proportion of SINE families among the Sox2- and Brn2-binding sites in ESC and NPC compared with that in the human genome. **C** Same as (**B**) except showing only ancient SINE families. **D** Proportion of LINE clades among the Sox2- and Brn2-binding sites in ESC and NPC. **E** Same as (**D**) except showing only ancient LINE clades. **F**,** G** Proportion of superfamilies of LTR retrotransposons (**F**) and DNA transposons (**G**) among the Sox2- and Brn2-binding sites in ESC and NPC

We mapped reads from public ChIP-seq data for Sox2 in ESC and NPC [50] to the human genome assembly hg38. In general, the unique assignment of reads to TE loci is more limited with single-end reads than with paired-end reads due to the repetitive nature of TE sequences. To address this issue, only reads judged to be uniquely mapped based on our filtering criteria, including MAPQ, were used in this analysis (see Methods). The summits of ChIP-seq peaks, *i.e.*, the positions with the highest read enrichment within each peak region, were defined as Sox2 binding sites. We identified 21,756 Sox2-binding sites in ESC, of which 5,956 (27.4%) were located within TEs (Fig. 1A, Additional file 1: Table S1). In NPC, 17,132 (20.2%) of a total of 85,006 Sox2-binding sites were derived from TEs. Only 1,821 TE-derived Sox2-binding sites were shared between ESC and NPC (Fig. 1B). This limited overlap between the two cell types indicates that the binding sites within TEs change dramatically during differentiation from ESC to NPC, consistent with previous findings on global Sox2-binding dynamics [37, 44]. Similarly, analysis of public Brn2 ChIP-seq data [51] revealed that 1,586 binding sites (35.6% of a total of 4,461), defined by ChIP-seq peak summits were located within TEs (Additional file 1: Table S2).

The proportion of currently active TE families, such as Alu and L1 (LINE-1), among all Sox2-bound TEs, was very small (Fig. 1C, E). This likely reflects our stringent criteria requiring uniquely mapped reads; that is, most reads derived from young TEs were mapped to multiple loci and therefore excluded from our analysis. In contrast, the proportion of the incidence of Sox2 binding to old retrotransposon families was often higher than expected based on the occupation of the TEs in the human genome (Fig. 1D, F–H). For example, ERV1 elements occupy 3.1% of the human genome and comprise 2.2% of ChIP-seq input reads (see Methods), and the equivalent proportion of binding sites would be expected to originate from ERV1 sequences under a random distribution model. In ESC, however, the incidence of Sox2 binding to ERV1 was 8.7% (1,770 among 20,434 binding sites), 2.8–4.0 times higher than expected (Fig. 1G). Similarly, Sox2 binding was overrepresented in ancient SINEs such as AmnSINE1 and LF-SINE, and ancient LINEs such as the RTE family, in both ESC and NPC (Fig. 1D, F).

### Two-wave expansion of TEs bound by Sox2 and Brn2

**Fig. 2:**
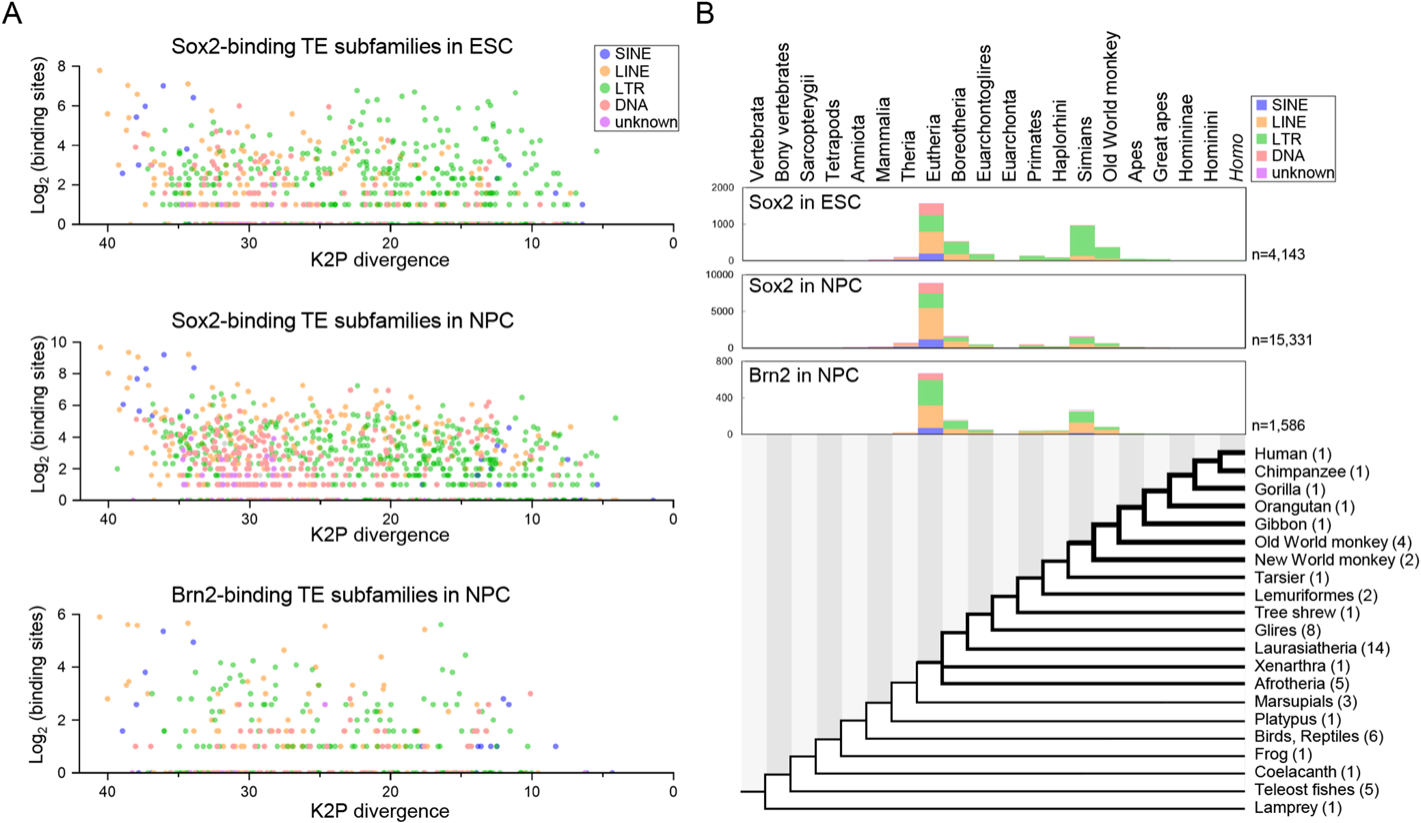
The evolutionary aspects of the Sox2- and Brn2-binding sites in ESC and NPC. **A** Log-scaled numbers of Sox2-binding TEs in ESC (top), Sox2-binding TEs in NPC (middle), and Brn2-binding TEs in NPC (bottom) for each TE family, plotted against the Kimura two-parameter (K2P) divergence of the corresponding TE families. The K2P divergence from the TE consensus sequence was averaged across all copies in the human genome and represents the relative age of (retro)transpositional activity. Plots are color-coded according to TE class. Note that the K2P divergence axes are inverted to align TE acquisition times with the phylogenetic tree shown in (**B**). **B** Evolutionary trace of the orthologous sequences of the human Sox2- and Brn2-binding TEs among vertebrates. Orthologous sequences of all human Sox2- and Brn2-binding sites located within TEs were searched across 60 vertebrate genomes using liftOver. The common ancestor of humans and the most distantly related species harboring the same ortholog was inferred to be the lineage in which the TE was inserted. Phylogenetic relationships were based on previous reports [78, 79], taking into account the polytomy of the last common ancestor of eutherians [80, 81]. The bar graphs indicate the number of binding sites for phylogenetic clades in which orthologs were detected in the most distantly related species, thereby inferring the timing of TE acquisition harboring these binding sites. TE classes are color-coded. Numbers in parentheses following each clade name in the phylogenetic tree indicate the number of species used for ortholog searches. Clade names are shown above the graphs

We next investigated when the TE sequences bound by Sox2 and Brn2 were (retro-)transposed during vertebrate evolution. The number of Sox2- and Brn2-binding sites in each TE subfamily were compared with relative timing of (retro-)transposition, estimated from the mean Kimura 2-parameter (K2P) divergence from the consensus sequence (Fig. 2A). In ESC, younger TEs with K2P divergence values of 10–20 were predominantly LTR retrotransposons, mainly belonging to the ERV1 family (Fig. 2A top). In contrast, in NPC, the contribution of younger TEs was relatively small whereas a variety of older TE classes with higher K2P divergence values contributed to Sox2-binding sites (Fig. 2A middle). Although the total number of Brn2-binding TEs in NPCs was limited, both ancient and relatively young TEs were represented (Fig. 2A bottom).

Furthermore, using liftOver and pairwise genomic alignments for human against each of the 60 species, we identified the most distantly related species in which orthologous sequences of the Sox2- and Brn2-binding sites were detected. In ESC, 37.9% of the TEs bound by Sox2 originated in the common ancestor of Eutheria (including all placentals), and the corresponding percentage for NPC was 57.9%. TEs acquired in the common ancestor of simians constituted 23.6% in ESC but only 10.1% in NPC (Fig. 2B). Among the Brn2-binding TEs, 46.3% and 21.5% were acquired in the common ancestors of Eutheria and simians, respectively. Thus, both Sox2-binding TEs in ESC and the Brn2-binding TEs in NPC exhibited a similar two-wave pattern of TE acquisition (Fig. 2B). This trend remained consistent even when the acquisition rate was normalized by the branch lengths between divergence times of the respective nodes, as obtained from TimeTree [52] (Additional file 2: Fig. S1A).

A comparable two-wave pattern of TE acquisition was previously observed for ERα-binding elements associated with mammary gland development, in which retrotransposition of ERV1 families produced numerous simian-specific ERα-binding sites in the human genome [31]. Our results revealed a similar pattern, in that simian-specific ERV families contributed to the genome-wide expansion of these binding sites. In particular, numerous ERV1 sequences serve as regulatory sequences in ESC [29, 53]. The expansion of ERV families in the common ancestor of simians may reflect either more frequent retrotransposition events or an increased probability of fixation due to a population bottleneck. Conversely, although certain ERV1 families have provided the Sox2-binding sites (Fig. 2A), most TEs bound by Sox2 in NPC are evolutionarily older than those in ESC (Fig. 2B).

Regarding non-TE sequences, the presence of orthologous sequences of Sox2-binding sites in NPC can be traced back to the eutherian common ancestor for 37.1% of binding instances and to earlier lineages for 61.1% (Additional file 2: Fig. S1A). Similarly, the existence of 46.3% and 44.3% of non-TE Brn2-binding sites could be traced back to a eutherian common ancestor and earlier, respectively. Overall, 98.2% and 90.6% of non-TE Sox2- and Brn2-binding sequences in NPC, respectively, were already present in the eutherian ancestor, suggesting that the molecular basis, *i.e.*, regulatory system, for neuronal differentiation was largely established in early eutherians. In contrast, because 93.0% and 97.4% of the Sox2- and Brn2-binding TEs, respectively, were acquired in and after the common ancestor of eutherians, implying that these TEs contributed to the expansion, rewiring, and fine-tuning of preexisting regulatory systems for neuronal differentiation during eutherian evolution. Notably, we identified more than 3,261 Sox2-binding TEs and 579 Brn2-binding TEs that emerged in or after the primate lineage which may hold important clues to the molecular mechanisms of primate-specific neuronal differentiation.

We further evaluated the evolutionary conservation of Sox2-binding sites based on average phastCons scores and found that TE-derived binding sites were slightly more conserved than their flanking regions in both ESC and NPC (*p* < 0.0001 by two-sided Welch’s t-test; Additional file 2: Fig. S1B). These results suggest that the TE-derived binding regions have been subject to weak purifying selection, indicating a certain degree of functional constraint. Non-TE Sox2-binding sites showed higher conservation, consistent with their origin in or before the eutherian ancestor (*p* < 0.0001 by two-sided Welch’s t-test; Additional file 2: Fig. S1A).

### Enrichment of Sox2- and Brn2-binding sites within TEs

We assessed the enrichment of Sox2-binding sites across 590 TE families in the human genome, using the distribution of ChIP-seq input reads as a background control (Additional file 2: Figs. S2, S3A-B, Additional file 1: Tables S3–S4). In ESC, 32 families showed significant enrichment of the Sox2-binding sites (*p* < 0.001; binomial test with Bonferroni correction). In NPC, significant enrichment was evident in three SINE families, three LINE families, 13 LTR-type retrotransposons, and five DNA transposons. Of these, five LTR-type retrotransposons (MER41, MER51, LTR9, LTR10, LTR26), and two DNA transposons (MER91, MER126) were significantly enriched in both cell types. These findings are consistent with previous findings that Sox2 binding was frequently observed in LTR retrotransposons in mouse ESC [27] and human induced pluripotent stem cells (iPSC) [54]. Notably, MER130, previously identified as a developmental enhancer in the mouse neocortex [20, 23], was also enriched for Sox2-binding sites in NPC, supporting their possible role as neuronal enhancers.

**Fig. 3:**
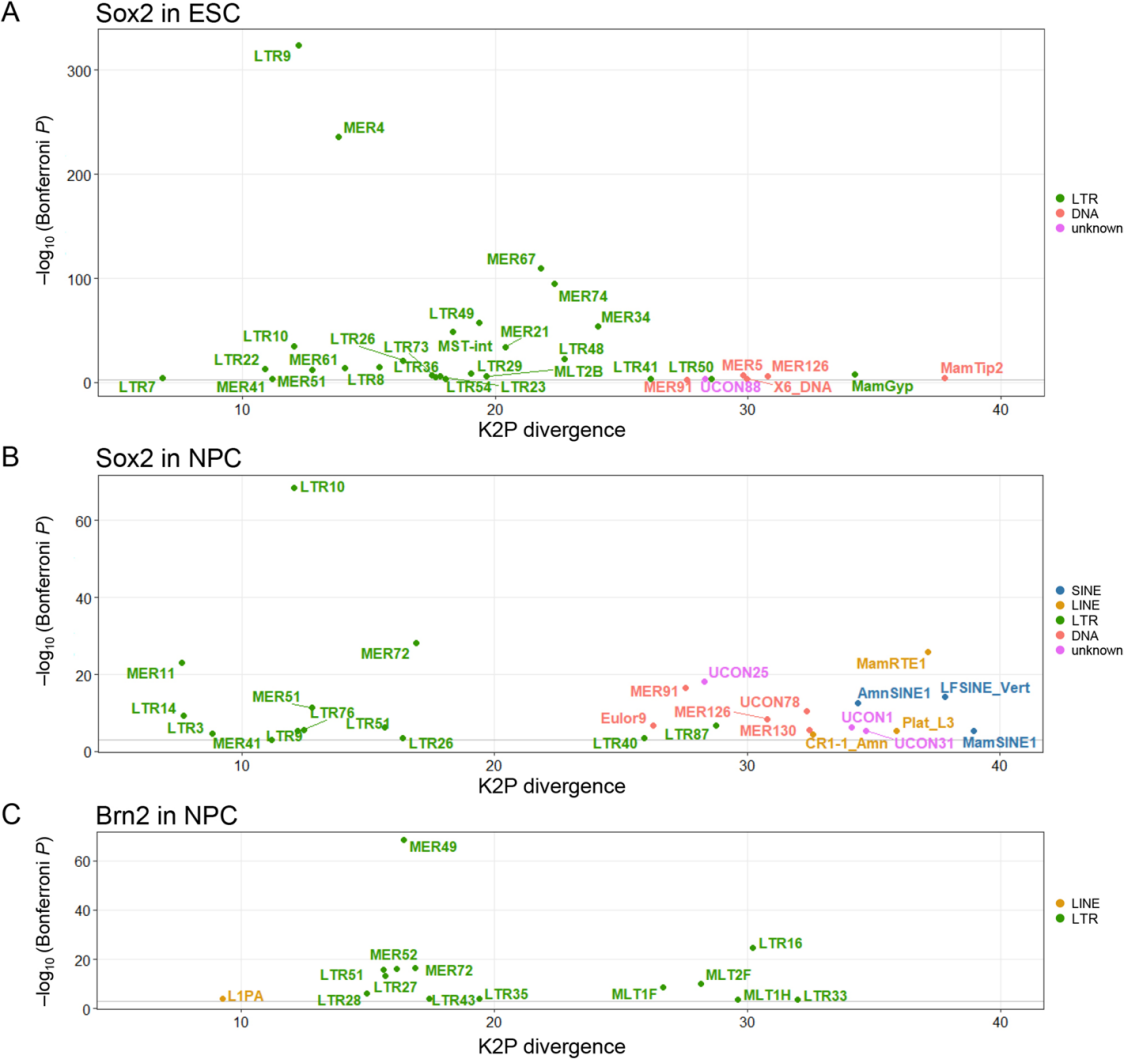
**A**–**C** TE families significantly enriched for Sox2 binding in ESC (**A**), Sox2 in NPC (**B**), and Brn2 in NPC (**C**) and their relative age of (retro-)transpositional activity. Binding-site enrichment within TEs was assessed by the binomial test (Bonferroni-adjusted *p* < 0.001), and log-scaled *p*-values are plotted against the mean Kimura two-parameter (K2P) divergence from the consensus sequence for each TE family

We next evaluated the relative ages of TE families based on their mean K2P divergence from the consensus sequence (Fig. 3, Additional file 2: Fig. S3C). LTR retrotransposons enriched for Sox2 binding in ESC were relatively young (K2P = 10–20) (Fig. 3A), consistent with the presence of primate-specific LTRs that function as *cis*-regulatory elements in human ESC [29, 53]. By contrast, SINEs and DNA transposons enriched for Sox2 binding in NPC were evolutionarily older (K2P > 25) (Fig. 3B), in line with the estimated timing of acquisition (Fig. 2). For instance, AmnSINE1 originated before the last common ancestor of Amniota [16, 21, 55, 56], and LF-SINEs can be traced back to the common ancestor of Sarcopterygii [15], suggesting that Sox2 binding to these ancient SINEs during neuronal differentiation might have occurred early in evolutionary time. These results suggest that the Sox2-binding landscape in TEs changes dramatically during ESC-to-NPC differentiation (Fig. 1B), with Sox2 bound to ERVs in ESC being released and subsequently rebinding to ancient TEs and other ERV families in NPC (Figs. 2A, 3A, B).

In NPC, Brn2-binding sites were enriched in 14 TE families, 13 (93%) of which belong to LTR retrotransposons (Fig. 3C, Additional file 2: Fig. S2C). Two LTR retrotransposons (MER72, LTR51) showed enrichment for both Sox2 and Brn2 bindings in NPC. Unlike Sox2, the two-wave acquisition of Brn2-binding TEs involved both young and ancient LTR families (Fig. 3C, Additional file 2: Fig. S3C). For example, the relatively young MER49 family showed the highest enrichment, whereas ancient MLT-related families were also significantly enriched for Brn2-binding sites. This pattern suggests that LTR retrotransposons have continuously supplied Brn2-binding sites throughout mammalian evolution.

This conclusion is further highlighted by analyses at the TE subfamily level. In ESCs, Sox2-binding sites were significantly enriched in 59 TE subfamilies, of which 51 (86%) belong to LTR retrotransposons with relatively low K2P divergence values (Additional file 2: Fig. S4A, D). In NPC, a distinct dual contribution of young LTRs and ancient other classes of TEs to the formation of Sox2-binding sites was observed (Additional file 2: Fig. S4B, E). Brn2-binding sites were enriched across both young and old LTR retrotransposons, as well as several L1 subfamilies (Additional file 2: Fig. S4C, F). The enrichment of Brn2 at the 5' promoter regions of older L1s may reflect its involvement in L1 transcription, consistent with previous reports that active human L1s can undergo retrotransposition in neurons [57–59].

### Evolutionary expansion of transcription factor–binding sites via retrotransposition

**Fig. 4:**
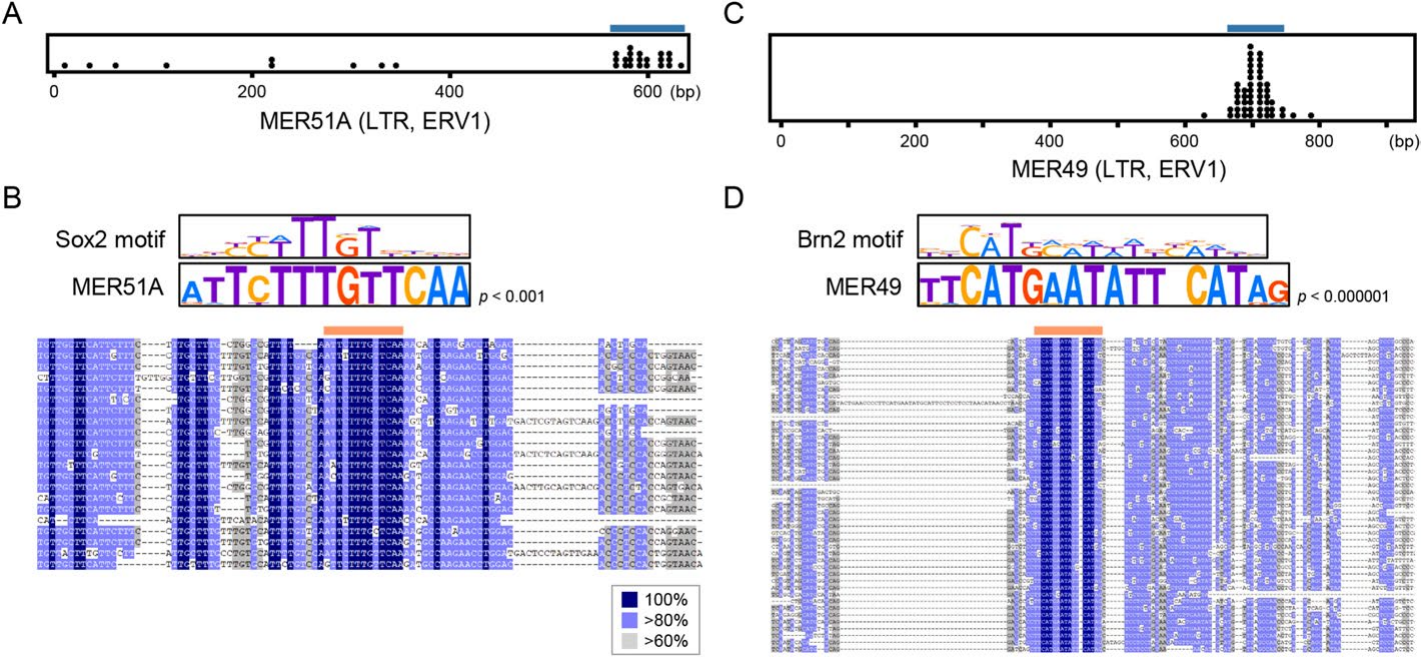
Two ERV1 families that had propagated the transcription factor–binding sites via retrotransposition. **A** The Sox2-binding sites in NPC were plotted along with the consensus sequence of MER51A, a simian-specific ERV1 family. The 21 Sox2-binding loci denoted by the horizontal blue line were used for the alignment in (**B**). **B** Alignment of the MER51A sequences containing the biased Sox2-binding sites as denoted by the blue line in (**A**). The sequence motif of the Sox2-binding region denoted by the orange line above the alignment was compared with the known Sox2-binding motif SOX2_HUMAN.H11MO.0.A obtained from the HOCOMOCO database [69]. **C** The Brn2-binding sites in NPC were plotted along with the consensus sequence of MER49, a Haplorrhini-specific ERV1 family. The 46 Brn2-binding sequences denoted by the horizontal blue line were used for the alignment in (**D**). **D** Alignment of the MER49 sequences with the biased Brn2-binding sites as denoted by the blue line in (**C**). The sequence motif of the Brn2-binding region denoted by the orange line above the alignment was compared with the known Brn2-binding motif PO3F2_HUMAN.H11MO.0.A obtained from HOCOMOCO

Given the significant enrichment for Sox2-binding sites in many TE families, we hypothesized that certain retrotransposons originally carried Sox2-binding motifs that were propagated through retrotransposition during evolution. If so, the Sox2-binding sites would be expected to be concentrated at specific positions within the TE consensus sequence. To test this, the genomic positions of Sox2-binding sites in TEs were converted to positions in the TE consensus sequence obtained from the RepeatMasker library (Dfam 3.6 integrated with Repbase ver. 20,181,026). In NPC, among the 1,180 MER51A LTR copies that occupy 0.013% of the human genome, Sox2 binds to 30 copies, showing significant enrichment (Bonferroni-corrected *p* < 0.001). Notably, 21 binding sites were concentrated near position 590 in the consensus sequence (634 bp full length; Fig. 4A), where a conserved motif in positions 587–599 matched the canonical Sox2 motif (*p* < 0.001 with FIMO analysis; Fig. 4B). Similar potential clustering was observed in three related subfamilies (MER51B/C/E), all sharing the Sox2 core motif 5'-CTTTGT-3' (Additional file 2: Fig. S5A–C). We also found a parallel pattern for Brn2. Within the MER49 family, which occupies 0.018% of the human genome, 50 out of 1,444 copies contain Brn2-binding sites (Bonferroni-corrected *p* < 10^–89^; Additional file 1: Table S3), concentrated around position 700 (Fig. 4C). A conserved sequence at positions 693–709 significantly matched the Brn2 motif (*p* < 10^–6^ with FIMO analysis; Fig. 4D). Both MER51A and MER49 belong to the ERV1 superfamily. MER51A is a simian-specific family, and MER49 is specific to Haplorrhini (simians and tarsiers), but they are currently retrotranspositionally inactive in the human genome. These findings suggest that, during the evolution of simian primates, MER51A and MER49 propagated and spread seed sequences capable of binding Sox2 and Brn2, some of which might have become sources of regulatory sequences serving in neuronal differentiation.

Extending this analysis to other enriched TE subfamilies (Fig. 3), we identified at least 22 TE subfamilies showing concentrated Sox2 or Brn2 binding sites within their consensus sequences (Additional file 2: Figs. S6–S8). Of these, 13 were bound by Sox2 in ESC, four by Sox2 in NPC, and five by Brn2 in NPC. Conserved motifs corresponding to Sox2 or Brn2 were detected in the regions with accumulated binding sites, and the TE consensus sequences contained the binding motifs (*p* < 0.01 for each; FIMO analysis). Collectively, these findings indicate that specific TE families have propagated potential seed sequences for transcription factor binding via retrotranspositions. These results support the Britten-Davidson model [60], in which repetitive elements could provide similar functions in the genome.

### Thousands of Sox2-binding TEs display enhancer-like chromatin signatures

**Fig. 5:**
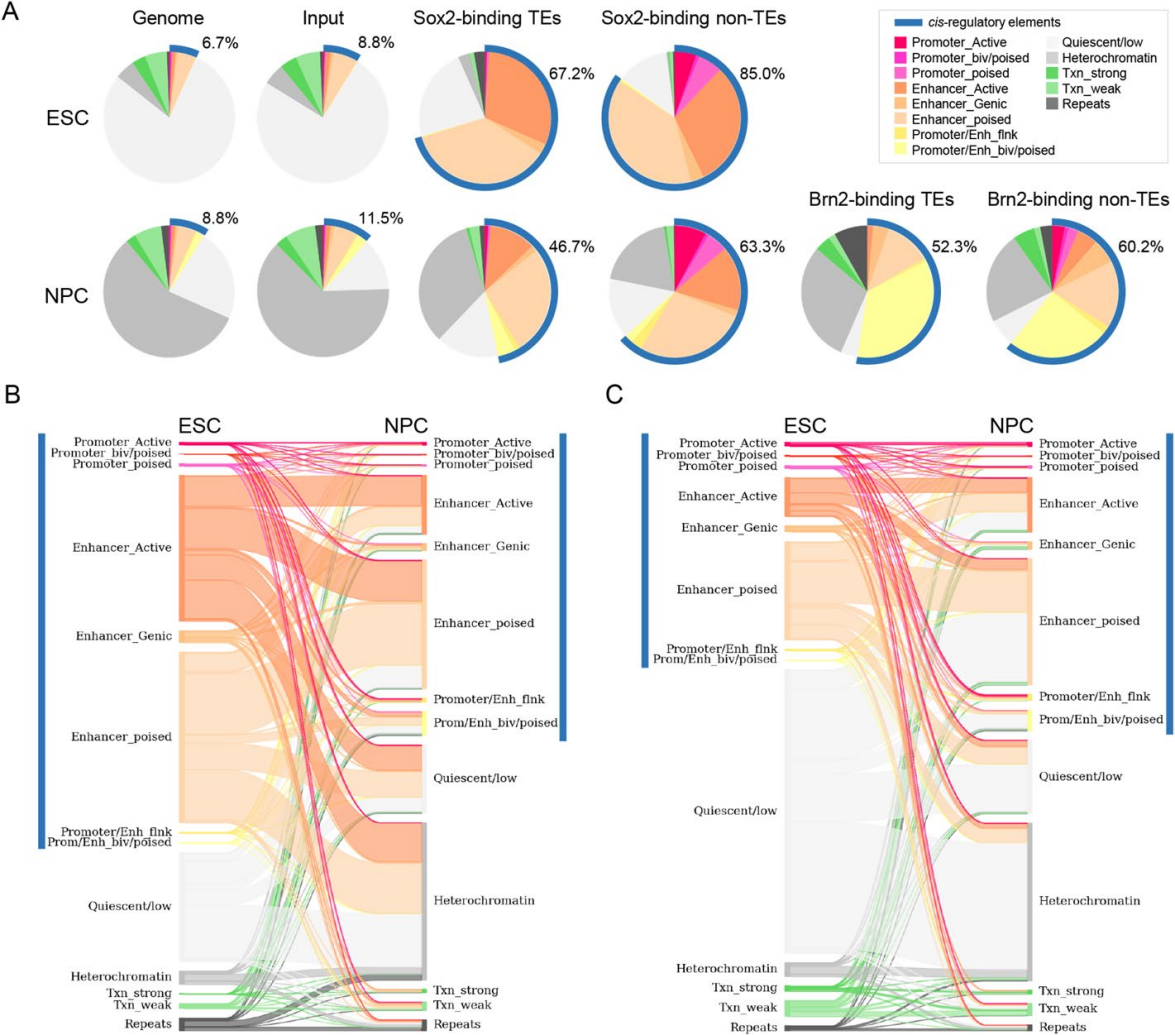
Functional annotation of the Sox2- and Brn2-binding sequences. **A** Fraction of the genomic functional categories in the human genome, random ChIP-seq input reads (control), and transcription factor–binding sites located in TE or non-TE sequences for ESC (top) and NPC (bottom). The functional annotations were estimated from histone modification patterns using the ChromHMM algorithm. The blue line along the circumference of each pie graph denotes *cis*-regulatory elements comprising promoters and enhancers, with their corresponding proportions (%) indicated. **B** Functional transitions of TEs showing ESC-specific Sox2 binding during the differentiation from ESC to NPC. The functions of the Sox2-binding TEs specific to ESC (left) were changed to the functional states in NPC (right), where Sox2 binding was not detected. **C** Same as (B) except that the TEs were bound by Sox2 in NPC but not in ESC. The functions of the Sox2-binding TEs in NPC (right) were derived from the functional states in ESC (left) in which Sox2 did not bind to the same TE sequences

A subset of TEs bound by Sox2 or Brn2 is expected to act as *cis*-regulatory elements such as promoters or enhancers [44]. To test this, we annotated chromatin states across the genome using ChromHMM and published histone modification data from ESC and NPC. *Cis*-regulatory elements accounted for 6.7% of the genome in ESC and 8.8% in NPC, proportions comparable to those observed for the distribution of ChIP-seq input reads used as controls (Fig. 5A; Additional file 1: Table S5). Among 5,956 TE-derived Sox2-binding sites in ESC, 4,002 (67.2%) overlapped *cis*-regulatory elements (*p* < 0.0001; two-sided binomial test), and 8,003 of 17,132 Sox2-binding TEs in NPC (46.7%) showed similar overlap (*p* < 0.0001). Indeed, these loci exhibited enrichment of H3K4me1 and H3K27ac histone modifications, which are typically associated with active enhancers, as well as high chromatin accessibility (Additional file 2: Fig. S9). Likewise, 52.3% of Brn2-binding TEs (831 of 1,537) were located within *cis*-regulatory elements (*p* < 0.0001). Overall, approximately half of all Sox2- or Brn2-binding TEs displayed enhancer-like chromatin features, suggesting that roughly 9,000 TEs may play gene regulatory roles during neuronal differentiation.

**Fig. 6:**
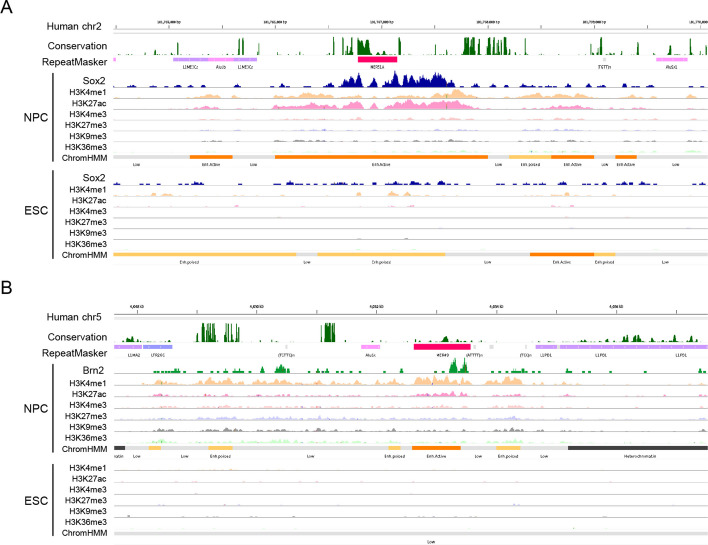
Instances of (**A**) Sox2-binding MER51A (chr2:101,766,784–101,767,143; hg38) and (**B**) Brn2-binding MER49 (chr5:4,052,637–4,053,571; hg38) loci. The upper window of the IGV browser represents genomic positions in the human genome assembly hg38, evolutionary conservation from phastCons30way (dark green), and TE annotations from RepeatMasker. The magenta bar in the RepeatMasker track indicates TEs in which ChIP-seq peaks of Sox2 (blue in **A**) and Brn2 (green in **B**) were detected specific to NPC. In each panels, histone modifications and functional annotations from ChromHMM are shown in the middle and bottom of the window, highlighting Active Enhancer functions (orange bars) of the TE sequences

Representative elements showed consistent patterns: 40.0% of Sox2-binding MER51A and 20.0% of Brn2-binding MER49 copies were located within *cis*-regulatory elements (Additional file 2: Fig. S5D). Indeed, MER51A and MER49 instances bound by Sox2 and Brn2, respectively, are located within active enhancer regions, exhibiting elevated H3K4me1 and H3K27ac levels in NPC relative to ESC (Fig. 6). Similarly, instances from 22 TE subfamilies that might have propagated transcription factor binding sites (Additional file 2: Figs. S6–S8) were also located within enhancer-annotated regions (Additional file 2: Figs. S10–S12).

We next examined dynamic changes in TE-associated regulatory activity during ESC-to-NPC differentiation. Among the ESC-specific Sox2-binding TEs, the proportion overlapping *cis*-regulatory regions decreased from 67.2% to 41.0% after differentiation into NPC (Fig. 5B), suggesting that 26.2% lost the *cis*-regulatory activity after loss of Sox2 binding. Notably, 42.8% of Sox2-binding enhancer-like TEs in ESC transitioned to quiescent or heterochromatin state in NPC (Fig. 5B). Conversely, 46.7% of NPC-specific Sox2-binding TEs exhibited chromatin states indicative of *cis*-regulatory activity in NPC (Fig. 5A), nearly half of which (48.3%) were inactive in the absence of Sox2 binding in ESC (Fig. 5C). In total, 2,737 TE loci gained both Sox2 binding and enhancer function after differentiation to NPC. These results suggest that thousands of TEs undergo functional transitions in conjunction with Sox2 binding during neural cell differentiation.

### Coupled expressional elevation of the nearest genes of the Sox2-binding TEs

**Fig. 7:**
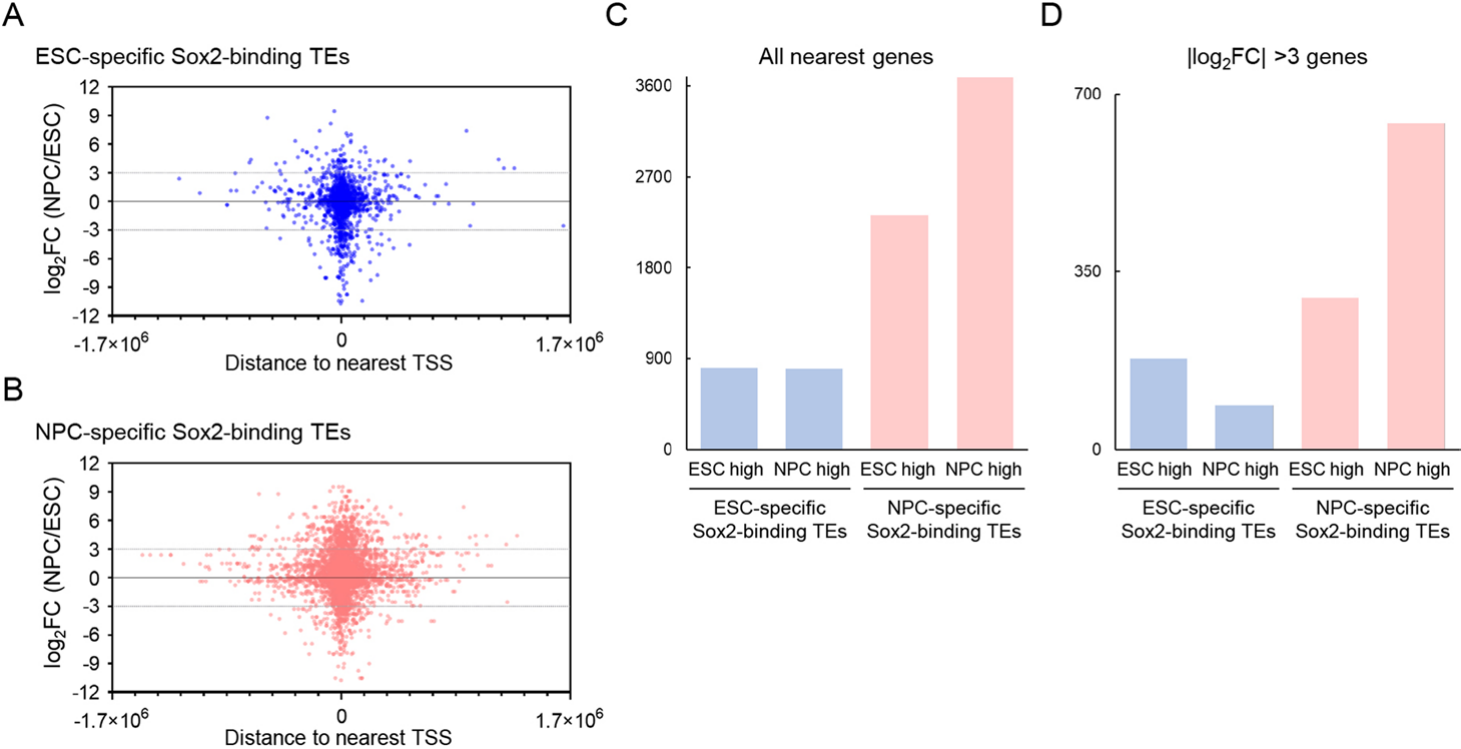
Changes in the expression levels of the nearest genes to Sox2-binding TEs during ESC-to-NPC differentiation. **A**, **B** Scatter plots showing the log2 fold change (FC) in gene expression (NPC/ESC ratio) for each gene and the genomic distance of Sox2-binding TEs from the transcription start sites (TSS) of those genes. The nearest genes to Sox2-binding TEs specific to ESC (**A**) and NPC (**B**) were analyzed. A distance < 0 indicates that the TE is located upstream of the TSS relative to the direction of transcription, and vice versa. The black horizontal line indicates no change, and the gray lines at log_2_FC of 3 and –3 represent eightfold upregulation and 1/eightfold downregulation, respectively. **C** Numbers of upregulated and downregulated genes during ESC-to-NPC differentiation whose TSSs are nearest to TEs bound by Sox2 specifically in ESC (light blue) and NPC (light magenta). "NPC high" indicates higher expression in NPC, and "ESC high" indicates higher expression in ESC than NPC. **D** Same as (**C**), except that only genes showing > eightfold or < 1/eightfold changes in expression are considered

To assess the transcriptional effects of Sox2-binding TEs, we examined expression changes in their nearest genes during ESC-to-NPC differentiation using published transcriptome data [50]. In both ESC and NPC, genes harboring Sox2-bound TEs near their transcription start sites (TSSs) exhibited larger expression changes than those with more distal binding (Fig. 7A, B). Notably, for NPC-specific Sox2-binding TEs, upregulated nearest genes outnumbered downregulated ones by 1.6-fold (3,685 vs. 2,324; Fig. 7C). When restricted to genes showing strong differential expression (> 8- or < 1/eightfold change), the imbalance increased to 2.1-fold (643 vs. 300; Fig. 7D). For ESC-specific Sox2-binding TEs, although the numbers of up- and downregulated nearest genes were roughly equivalent, highly upregulated genes in ESC (FC > 8) were twice as common as those with FC < 1/8 (Fig. 7C, D).

Furthermore, we next performed gene ontology analysis using GREAT [61] for NPC-specific Sox2-binding TEs whose nearest genes were upregulated in NPC (Additional file 2: Fig. S13). GO Biological Process terms with significant enrichment did not reveal any distinctive terms, and the other GO domains (Cellular Component and Molecular Function) showed no clear enrichment. Remarkably, however, the phenotype ontology of knock-out mice revealed significant associations with abnormalities in the nervous system, hippocampus, and somatosensory cortex (Additional file 2: Fig. S13B, C) [62]. These findings suggest that genes located near NPC-specific Sox2-binding TEs are functionally involved in neurogenesis and brain development.

In this study, we focused exclusively on the enhancer/promoter functions of Sox2-binding sites within TEs. However, Sox2 is also known to participate in transcriptional repression by forming repressor complexes [50]. A subset of Sox2-bound TEs identified in this study did not exhibit chromatin states associated with *cis*-regulatory activity but were located in regions with low epigenetic signals or within heterochromatin (Fig. 5A), suggesting a potential role in gene silencing. On the other hand, Sox2 has also been reported to bind to the promoters of young L1 elements and to be involved in their transcription, while a decrease in Sox2 in NPC is correlated with L1 activation [57, 63]. However, we did not examine such young TEs in this study, as our stringent criterion of using uniquely mapped ChIP-seq reads likely led to a substantial underestimation of transcription factor binding events at these young TEs (see Methods). Future studies aimed at elucidating the full landscape of Sox2-mediated regulation across both young and old TEs during neural differentiation will be crucial for a deeper understanding of gene regulatory dynamics.

## Conclusions

Our study uncovered tens of thousands of TEs bound by Sox2 and Brn2 in human NPC, illuminating their evolutionary origins and functional transitions in conjunction with Sox2-binding patterns during neural cell differentiation. Approximately 8,000 Sox2-binding TEs exhibited chromatin states specific to *cis*-regulatory activity in NPC, twice as many as in ESC, with particularly strong contributions from TEs inserted in or after the eutherian ancestor. At least 24 TE families have contributed to the genome-wide propagation of Sox2 and Brn2 binding motifs, as supported by: (1) biased distribution of binding sites within TEs, (2) the presence of binding motifs in the TE consensus sequences, (3) motif conservation among TE copies, and (4) TE instances exhibiting enhancer-specific epigenetic signatures.

Orthologs of non-TE binding sites could be traced back to reptiles and fishes, suggests that the core regulatory framework for neuronal differentiation predates the eutherian ancestor. Subsequent lineage-specific retrotranspositions of TEs, particularly those of the ERV1 families, expanded Sox2- and Brn2-binding *cis*-regulatory repertoires during simian evolution, yielding over 3,000 Sox2-binding and 500 Brn2-binding sites in NPC. A subset of TE families, including MER51A and MER49, substantially contributed to this expansion by propagating Sox2 and Brn2 motifs across the genome, consistent with the Britten-Davidson model [60]. Unlike mice, lack of Brn2 causes a malformed phenotype in telencephalon leading to a lethal outcome in primates [47], suggesting an involvement in the primate-specific downstream regulatory system. TE-mediated enhancer acquisition may have diversified the *cis*-regulatory landscape underlying neuronal formation in simian primates.

Finally, by capturing functional transitions of TE-derived regulatory elements coupled with a dramatic change in Sox2-binding patterns across ESC-to-NPC differentiation, our findings highlight that co-opted/exapted TEs have been deeply involved in neuronal lineage commitment, which was largely unknown. This also implies that analyses limited to single tissues/cell types may overlook many co-opted/exapted TEs among 4.5 million copies of human TEs. Broader exploration of TE functional activity across tissues and conditions will be essential to reveal how transposition has shaped mammalian—and particularly human—brain evolution.

## Methods

### Identification of Sox2-binding sites from ChIP-seq data obtained for ESC and NPC

We used published Sox2 ChIP-seq data for human ESC and NPC [50] (Additional file 1: Table S6). Brn2 ChIP-seq data for human NPC were also obtained [51]. Each of the raw data were downloaded from the NCBI Sequence Read Archive using SRA Toolkit v3.0.0 and converted to fastq format using fasterq-dump. The raw reads were preprocessed with fastp ver. 0.23.2 [64] with default setting. All reads that passed the fastp quality check were mapped to the human genome assembly hg38 using Bowtie2 ver. 2.4.5 [65] with default parameters except for the "–very-sensitive-local" option. Unmapped reads were excluded using Sambamba ver. 0.6.6 [66] (-F "not unmapped") (Additional file 2: Fig. S14A). Mapped data with a MAPQ score of > 35 were selected to exclude multiply mapped reads (19.3% of reads; Additional file 2: Fig. S14B) with Sambamba ver. 0.6.6 [66] (using -F "mapping_quality > 35"). Reads from PCR duplicates were excluded using markdup of samtools ver. 1.13. The number of ChIP-seq reads retained or removed during the selection procedure are shown in Additional file 2: Fig. S14A. ChIP-seq peak call was conducted using MACS3 [67] with default parameters (–qvalue 0.05) except for the "–call-summits" option. The corresponding ChIP-seq input data for ESC and NPC were used as controls in the peak call.

To evaluate the validity of the MAPQ threshold above, we randomly extracted 1,000 reads for each MAPQ score between 11 and 40 from the mapped data using an in-house Perl script. A local BLASTn search was conducted against the human genome (hg38) with the "-word_size 11 -dust no -evalue 0.1" option, and we evaluated whether the BLAST search produced a top hit, with the higher BLAST Bit score than the second hit, corresponding to the locus mapped by Bowtie2. For the Bowtie2 mapping results with higher MAPQ score, the BLAST search reproduced the identification of the same locus (Additional file 2: Fig. S14C); *e.g.*, for the lowest MAPQ score of 36 in our threshold, 99.5% of the reads were mapped to the same locus between BLAST and Bowtie2. Therefore, most of the ChIP-seq reads selected in this study were uniquely mapped to the loci even if they are derived from repeat sequences.

We also evaluated the proportion of TEs for the selected or excluded ChIP-seq reads using the MAPQ score. Ten thousand Sox2 ChIP-seq reads in NPC were randomly selected for three MAPQ-score categories, *i.e.*, 1–10 (excluded), 11–35 (excluded), and 36–44 (used), and we investigated whether the start site of the Bowtie2-mapped locus corresponds to TEs (Additional file 2: Fig. S14D). Both MAPQ-score 1–10 and 11–35 categories includes over 80% of TE-derived reads, and the 1–10 category contain younger TE sequences indicated by lower divergence from their consensus sequences. In contrast, 35.8% of the ChIP-seq reads with the MAPQ score between 36 and 44, which we used in this study, are derived from repetitive elements (Additional file 2: Fig. S14E), most of which are anciently-integrated TE sequences with higher divergence (Additional file 2: Fig. S14D). Therefore, although retrotransposons propagate the identical sequences in the genome even in recent ages, accumulation of nucleotide substitutions in the TEs during evolution enabled us to identify a single TE locus. Rather, these stringent criteria may miss most reads derived from young TEs such as Alu and L1, leading to an underestimation of transcription factor binding events to these elements. Nevertheless, we adopted this approach to prioritize the more reliable detection of binding sites.

### Enrichment analysis of Sox2- and Brn2-binding sites in TE sequences

Repeat annotation was performed for the human genome assembly hg38 using RepeatMasker ver. 4.1.2-p1 (with the Dfam 3.6 repeat library; https://www.repeatmasker.org/RepeatMasker/) and Repbase repeat library ver. 20,181,026 [68] with the "-species human -e crossmatch -s -align -small" options. Sox2- and Brn2-binding sites overlapping with TEs were identified by comparing the summits of the ChIP-seq peaks with the TE location data in the RepeatMasker output. The binding sites in the hg38 genome were converted to the location of the consensus sequence of each TE family/subfamily by referring to the align file obtained from the RepeatMasker analysis. The number of ChIP-seq peak summits overlapping with TEs was counted for each of the 590 total TE families in the human genome. Mean Kimura two-parameter (K2P) divergence for each TE family was calculated from the K2P values of all TE copies in the RepeatMasker ".align" output. These analyses were conducted by using in-house Perl scripts.

To serve as a control for evaluating the enrichment of binding sites within TEs, the ChIP-seq input reads of Sox2 in NPCs were mapped to the human genome (hg38) and filtered as described above. From the uniquely mapped data, one million input reads were randomly selected, and their mapped positions were compared with the RepeatMasker output to determine the number of input reads originating from each of the 590 TE families.

The fold enrichment of binding sites in each TE family was calculated using the following formula:

$$Fold\;enrichment=\frac{\frac{Number\;of\;ChIPseq\;peak\;summits\;within\;the\;TE\;family}{Total\;number\;of\;ChIPseq\;peaks}}{\frac{Number\;of\;input\;reads\;mapped\;to\;the\;TE\;family}{Total\;number\;of\;input\;reads}}$$

The enrichment of Sox2 and Brn2 binding sites within TEs was evaluated using a two-sided binomial test, comparing the observed number of peaks with the mapping rate of input reads to the corresponding TE families (Additional file 1: Tables S3–S4). This analysis was performed in R (v4.4.0), and Bonferroni-adjusted *p*-values (*p* < 0.001) were used for significance assessment.

### Motif analysis for Sox2 and Brn2 binding sites in TEs

For the motif analysis, known Sox2- and Brn2-binding motifs (Sox2: SOX2_HUMAN.H11MO.0.A and SOX2_MOUSE.H11MO.0.A; Brn2: PO3F2_HUMAN.H11MO.0.A and PO3F2_MOUSE.H11MO.0.A) were obtained from the HOCOMOCO database [69]. Consensus sequences of TEs were retrieved from Repbase [68]. The presence of Sox2- and Brn2-binding motifs within TE consensus sequences was examined with FIMO (find individual motif occurrences) [70] with a significance threshold of *p* < 0.01 using the HOCOMOCO motifs. Multiple sequence alignments of TE copies were generated using MAFFT v7.495 [71] with the "–localpair –maxiterate 1000" option, and visualized with GeneDoc v2.6. TE loci in the human genome (hg38) were visualized with Integrative Genomics Viewer (IGV) [72].

### Functional annotation of the human genome by epigenetic signals

ChIP-seq data were obtained from the NCBI Sequence Read Archive for histone modifications H3K4me1, H3K4me3, H3K27ac, H3K27me3, H3K36me3, and H3K9me3 for ESC and NPC [73] (Additional file 1: Table S6). The reads were processed and mapped to the human genome assembly hg38 as described above. ChromHMM v1.25 [74] was used learn the epigenetic states and classify the genomes into 16 states (Additional file 2: Fig. S15). Evolutionary conservation scores (hg38.phastCons30way.bw) were obtained from the UCSC genome browser database [75]. Locations of TE classes (SINE, LINE, LTR, and DNA transposons) from the RepeatMasker output, transcription start and end sites, and the evolutionary conservation scores were also referenced to determine chromatin features. The functional annotations of all Sox2- and Brn2-binding sequences were assigned based on the chromatin features at the ChIP-seq peak summits identified by MACS3. As a control, functional annotations were also assigned to one million randomly selected positions from the input reads.

Raw ATAC-seq data for ESC and NPC [76] were obtained from NCBI SRA (Additional file 1: Table S6) and processed in the same manner as the ChIP-seq data for histone modifications. BigWig files were generated from the BAM files of ChIP-seq (Sox2, Brn2, and histone modifications) and ATAC-seq data using deepTools ver. 3.2.1 [77] with reads per kilobase per million mapped reads (RPKM) normalization (–normalizeUsing RPKM). Heatmaps of the signal intensities across TEs and their 1-kbp flanking regions were produced using computeMatrix with the options "reference-point –referencePoint center -b 1000 -a 1000 –missingDataAsZero –skipZeros" followed by visualization with plotHeatmap in deepTools ver. 3.2.1 [77].

### Ortholog identification for the Sox2-binding sites

Orthologous sequences for the Sox2- and Brn2-binding 10-bp sequences around the peak summits were searched using liftOver (https://genome.ucsc.edu/FAQ/FAQdownloads.html#liftOver). Chain data of the pairwise genome alignment between human and each of the 60 species were obtained from the UCSC Genome Browser [75] and utilized for the liftOver analysis (Additional file 1: Table S7). The clade specificity of all TE subfamilies was obtained from the RepeatMasker library, and the limitation of the evolutionary presence of TEs was taken into account to determine the presence or absence of an orthologous binding sequence in TEs for all loci. Phylogenetic relationships were based on previous reports [78, 79], taking into account the polytomy of the last common ancestor of eutherians [80, 81]. For each Sox2- and Brn2-binding locus, the most distantly related species containing the same ortholog were inferred based on these phylogenetic relationships. Median divergence times were retrieved from TimeTree (accessed on September 17, 2025) [52], and the rate of TE acquisition was calculated as the number of Sox2- and Brn2-binding TEs divided by the time span of each evolutionary branch.

### Evolutionary conservation

Evolutionary conservation of the Sox2-binding sequences was evaluated based on phastCons30way conservation scores for the human genome assembly hg38 obtained from the UCSC Genome Browser [75]. Average scores were calculated for the 2-kb region encompassing the Sox2-binding sites in the ESC and NPC genomes using deepTools ver. 3.2.1 [77]. The one million random input reads described above were classified as TE or non-TE based on the RepeatMasker annotation of their mapped positions, and conservation scores calculated in the same manner were used as background controls. Differences in the mean conservation scores between Sox2-binding sites and the mapped positions of control input reads were assessed using a two-sided Welch’s t-test.

### Transcriptome analysis for nearest genes to Sox2-binding TEs

For all TEs bound by Sox2 specifically in ESC and NPC, the nearest TSSs of RefSeq genes were identified by comparing the binding sites (ChIP-seq peak summits) with gene annotation data (hg38.ncbiRefSeq.gtf) obtained from the UCSC Genome Browser [75]. Gene expression data (RPKM) for ESC and NPC [50] were retrieved from NCBI GEO (GSE69476). Changes in expression levels between ESCs and NPCs were analyzed for the nearest genes of both ESC-specific and NPC-specific Sox2-binding TEs. Gene ontology analysis was performed using GREAT [61] with default parameters for NPC-specific Sox2-binding TEs whose nearest genes were upregulated in NPC.

## Supplementary Information

Additional file 1: Supplementary Tables S1–S7.

Additional file 2: Supplementary Figures S1–S15.

## Data Availability

Publicly available next-generation sequencing datasets were obtained from the NCBI Sequence Read Archive (Additional File 1: Table S6). The datasets used in this study are as follows: Sox2 ChIP-seq data in ESC and NPC (PRJNA285791) [50], Brn2 ChIP-seq data in NPC (PRJNA301599) [51], ChIP-seq data for histone modifications (H3K27ac, H3K27me3, H3K36me3, H3K4me1, H3K4me3, H3K9me3) in ESC and NPC (PRJNA34535) [73], and ATAC-seq data in ESC and NPC (PRJNA986429) [76]. Gene expression data for ESCs and NPCs were retrieved from the NCBI GEO (GSE69476) [50].
