# Supplementary material for: Transposable element–mediated evolutionary expansion of Sox2- and Brn2-binding regulatory modules for mammalian neural-cell differentiation
Source: Genome Biol. 2026 Apr 9;27:114. doi: 10.1186/s13059-026-04050-w (PMC13063735; doi:10.1186/s13059-026-04050-w)
Supplement: Supplementary file 2 — Additional file 2: Supplementary Figures S1–S15. [file 13059_2026_4050_MOESM2_ESM.pdf]

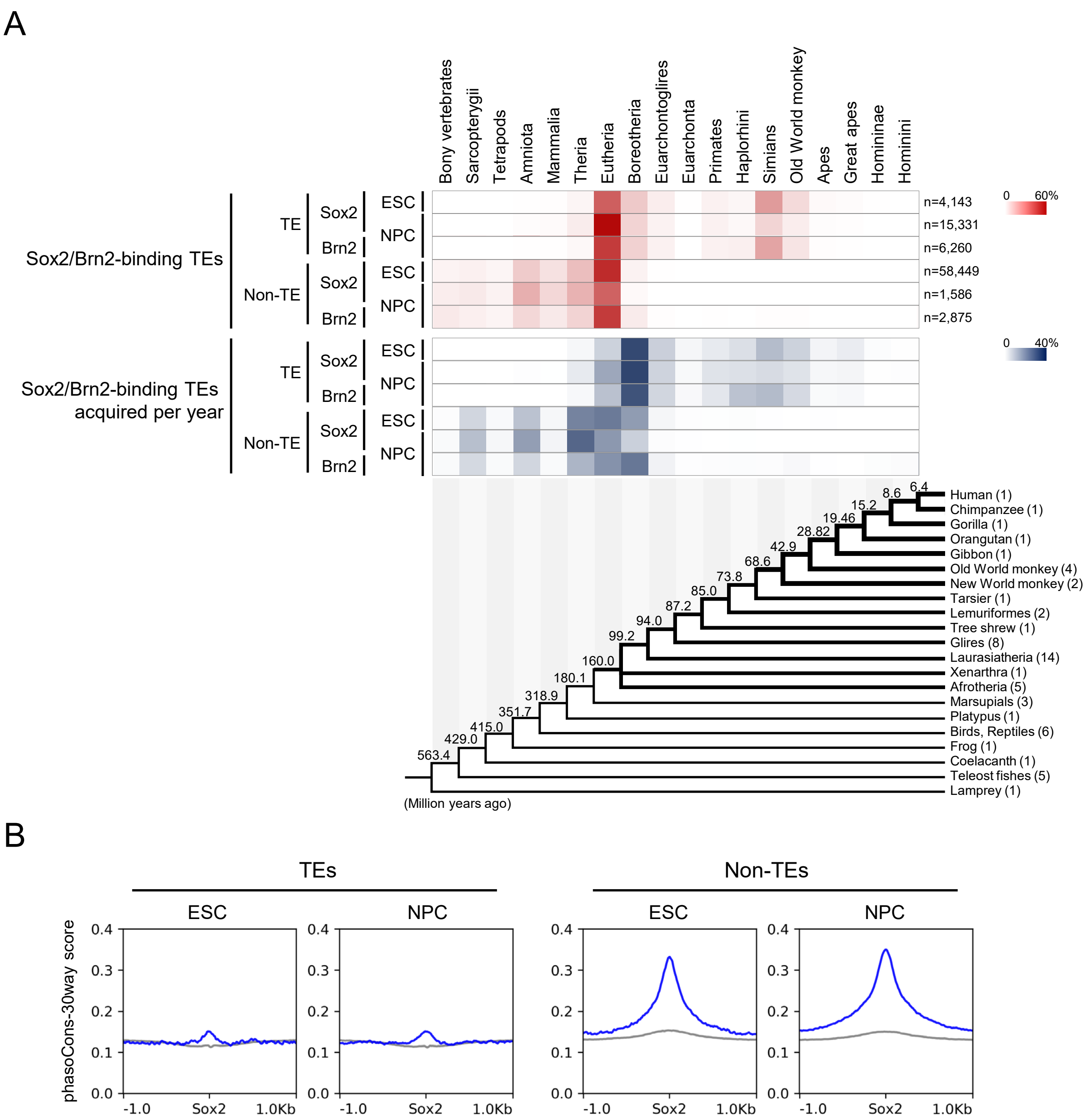

Fig. S1. The evolutionary aspect of the Sox2- and Brn2-binding sites in ESC and NPC. **A** Evolutionary trace of orthologous sequences of human Sox2- and Brn2-binding sites across vertebrates. The red heatmap denotes the proportion of binding sites among phylogenetic clades in which orthologs were detected in the most distantly related species, thereby inferring the temporal acquisition of TEs harboring these binding sites. The blue heatmap indicates the rate of acquisition of TE-derived binding sites per unit time. Divergence times (million years ago), as estimated by TimeTree (https://timetree.org/), are shown above the phylogeny. Numbers in parentheses following each species name indicate the number of species included in the ortholog search. Clade names are indicated above the heatmap. **B** Evolutionary conservation scores across 2-kb regions centered on Sox2-binding sites (blue) and random ChIP-seq input read positions (gray). Conservation was assessed as the mean phastCons30way score within the binding regions, calculated using deepTools.

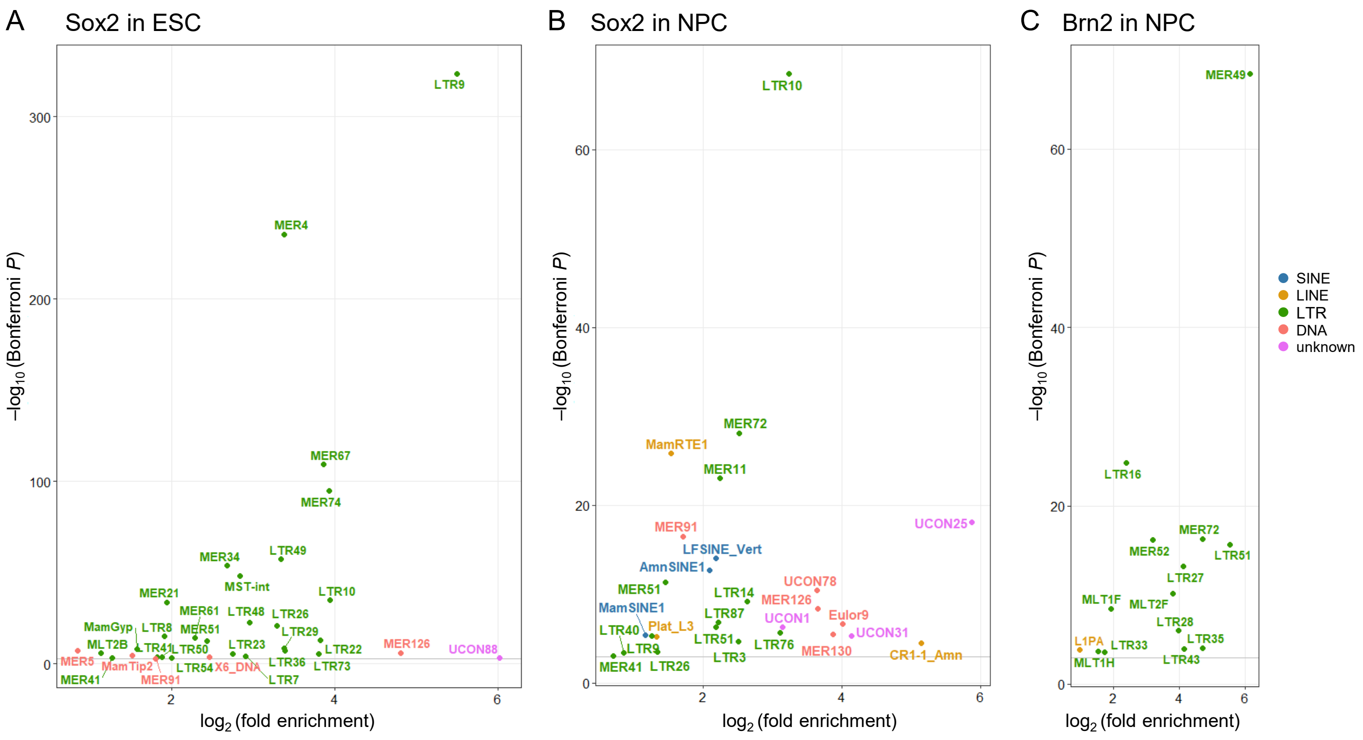

Fig. S2. A–C TE families significantly enriched for Sox2 binding in ESC (**A**), Sox2 in NPC (**B**), and Brn2 in NPC (**C**).Enrichment was assessed using a binomial test with Bonferroni correction ($p < 0.001$). Fold enrichment was calculated as the proportion of binding sites within each TE family relative to the expected proportion based on ChIP-seq input read distribution mapped to the TEs.

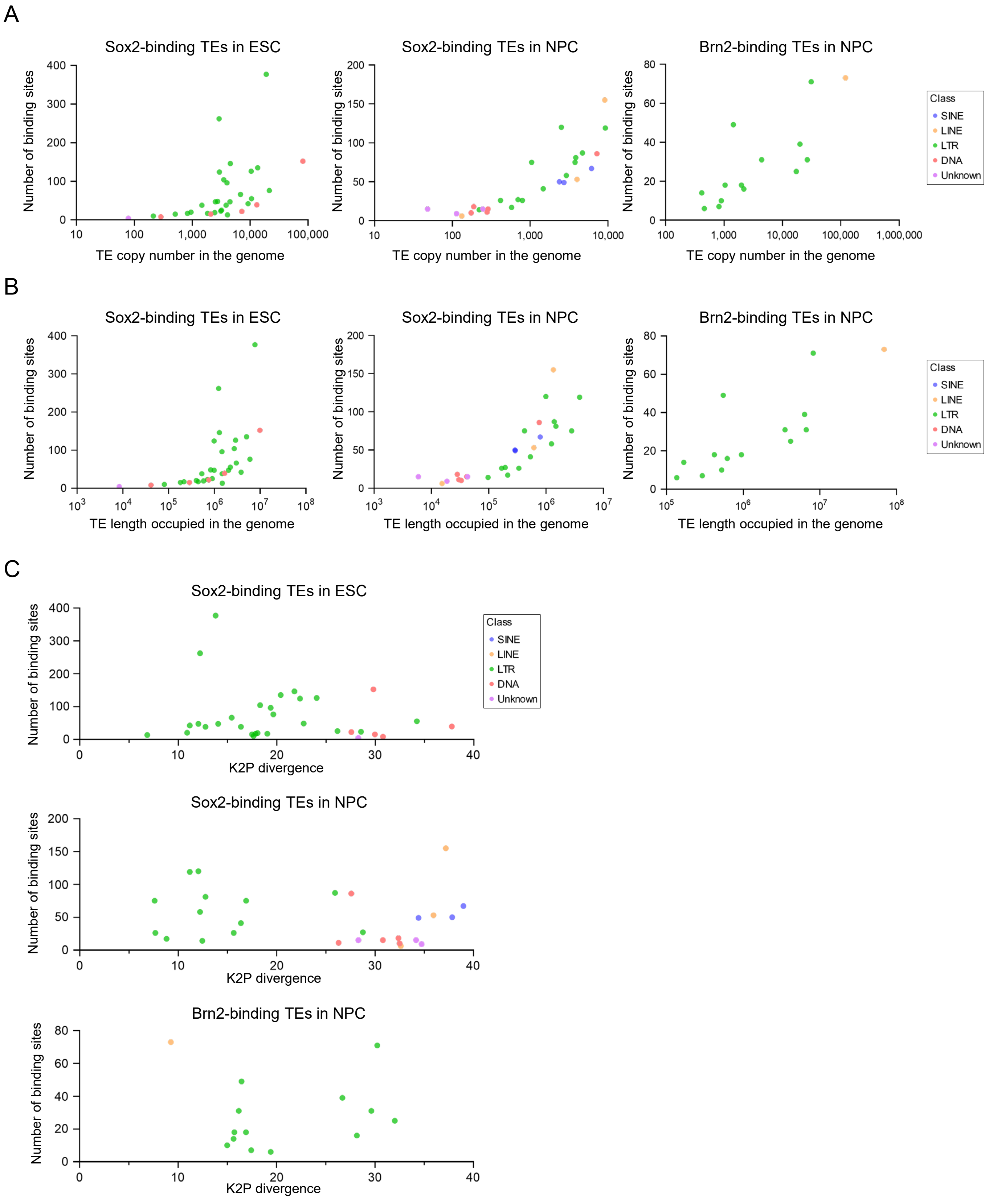

Fig. S3. Copy numbers of TE families showing significant enrichment of Sox2- and Brn2-binding sites in Fig. S2. **A** Numbers of TE copies bound by Sox2 or Brn2 compared with the total copy numbers of the corresponding TE families in the human genome. **B** Numbers of Sox2- or Brn2-bound TE copies relative to the total length of each TE family in the genome. **C** Numbers of Sox2- or Brn2-bound TE copies plotted against the mean Kimura 2-parameter (K2P) divergence of all copies from their consensus sequences in each TE family. In all panels, The TE families are color-coded according to their classes.

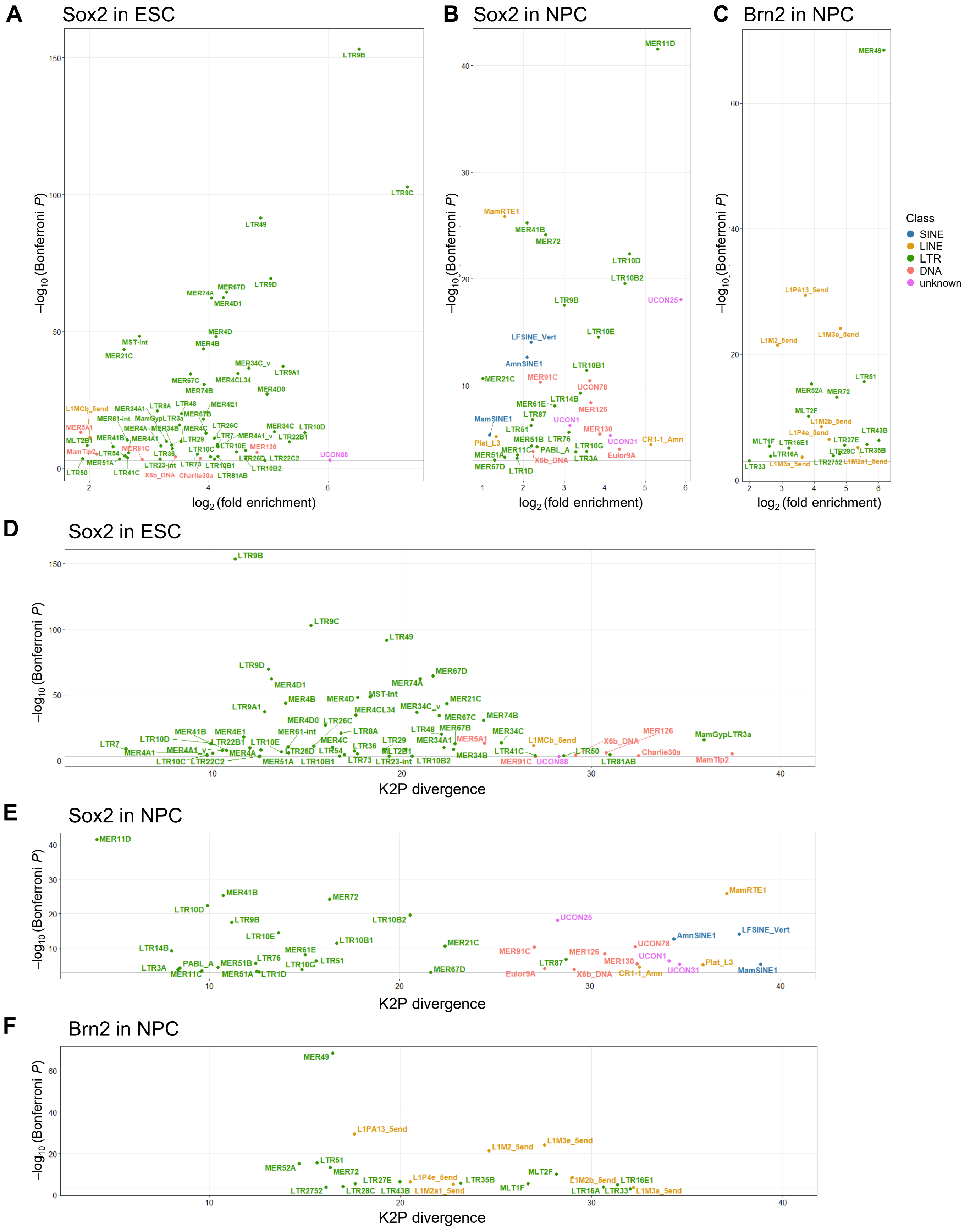

Fig. S4. Enrichment of Sox2- and Brn2-binding sites within TE subfamilies. **A–C** TE subfamilies significantly enriched for Sox2 binding in ESC (**A**), Sox2 in NPC (**B**), and Brn2 in NPC (**C**).Enrichment was assessed using a binomial test with Bonferroni correction (p < 0.001). Fold enrichment was calculated as the proportion of binding sites within each TE subfamily relative to the expected proportion based on ChIP-seq input read distribution mapped to the TEs. **D–F** The significantly enriched TE subfamilies and their relative age of (retro-)transpositional activity. Binding-site enrichment within TEs was assessed by the binomial test (Bonferroni-adjusted p < 0.001) , and log-scaled p-values are plotted against the mean Kimura two-parameter (K2P) divergence from the consensus sequence for each TE subfamily.

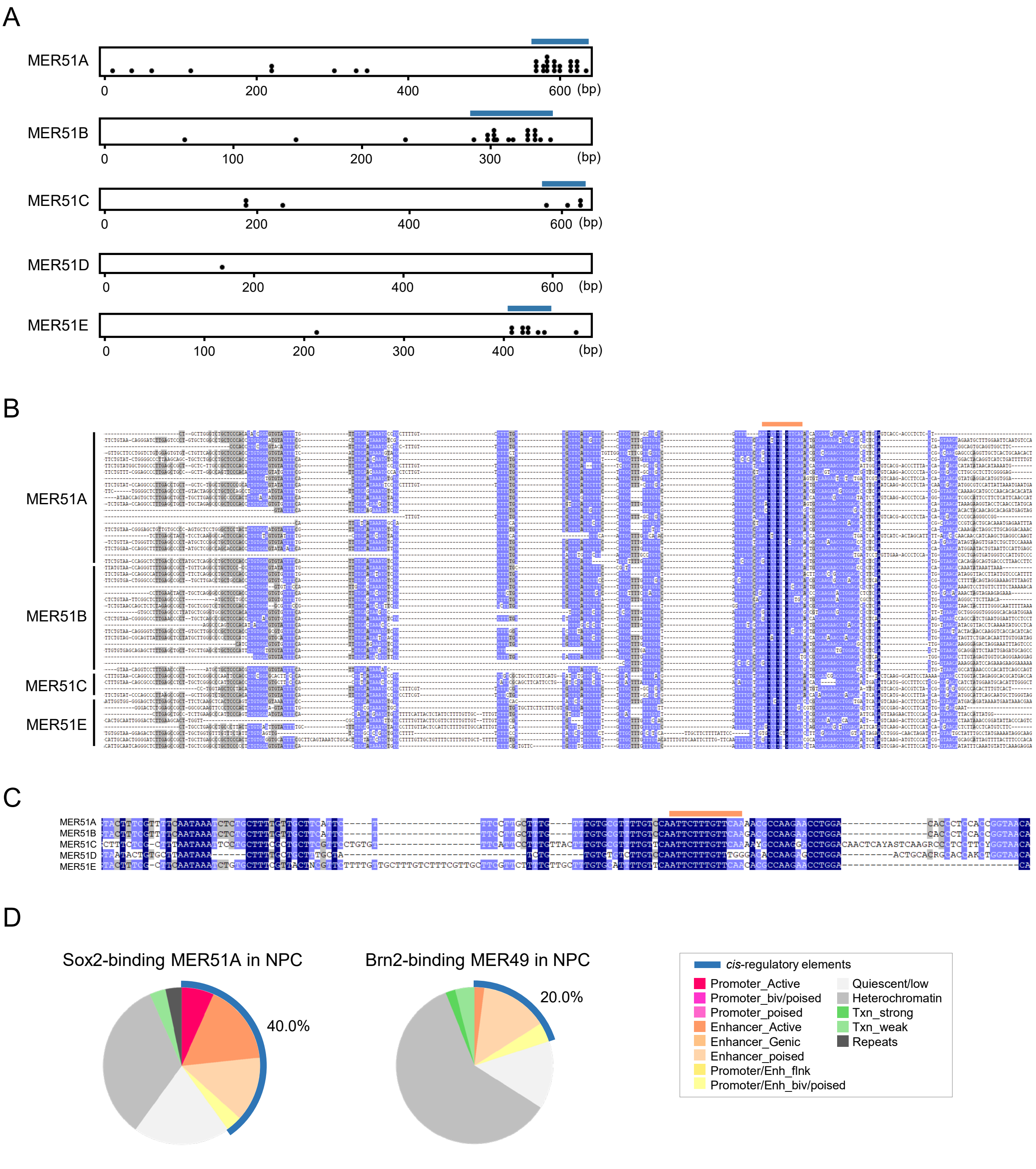

Fig. S5. MER51 subfamilies and functional estimation of the Sox2-binding MER51A and Brn2-binding MER49 elements in NPC. **A** The Sox2-binding sites in NPC were plotted along with the consensus sequences of the MER51A –MER51E subfamilies. **B** Alignment of the MER51A–MER51E sequences containing the biased Sox2-binding sites as denoted by the blue line in (A). The conserved sequence motif of the Sox2-binding region are denoted by the orange line above the alignment. **C** Alignment of the consensus sequences of the five MER51 subfamilies containing the shared Sox2-binding motifs denoted by the orange line. **D** Functional classification of the Sox2-binding sites in MER51A and Brn2-bnding sites in MER49 estimated by ChromHMM. *Cis*-regulatory elements composed of promoters and enhancers are denoted by the blue lines.

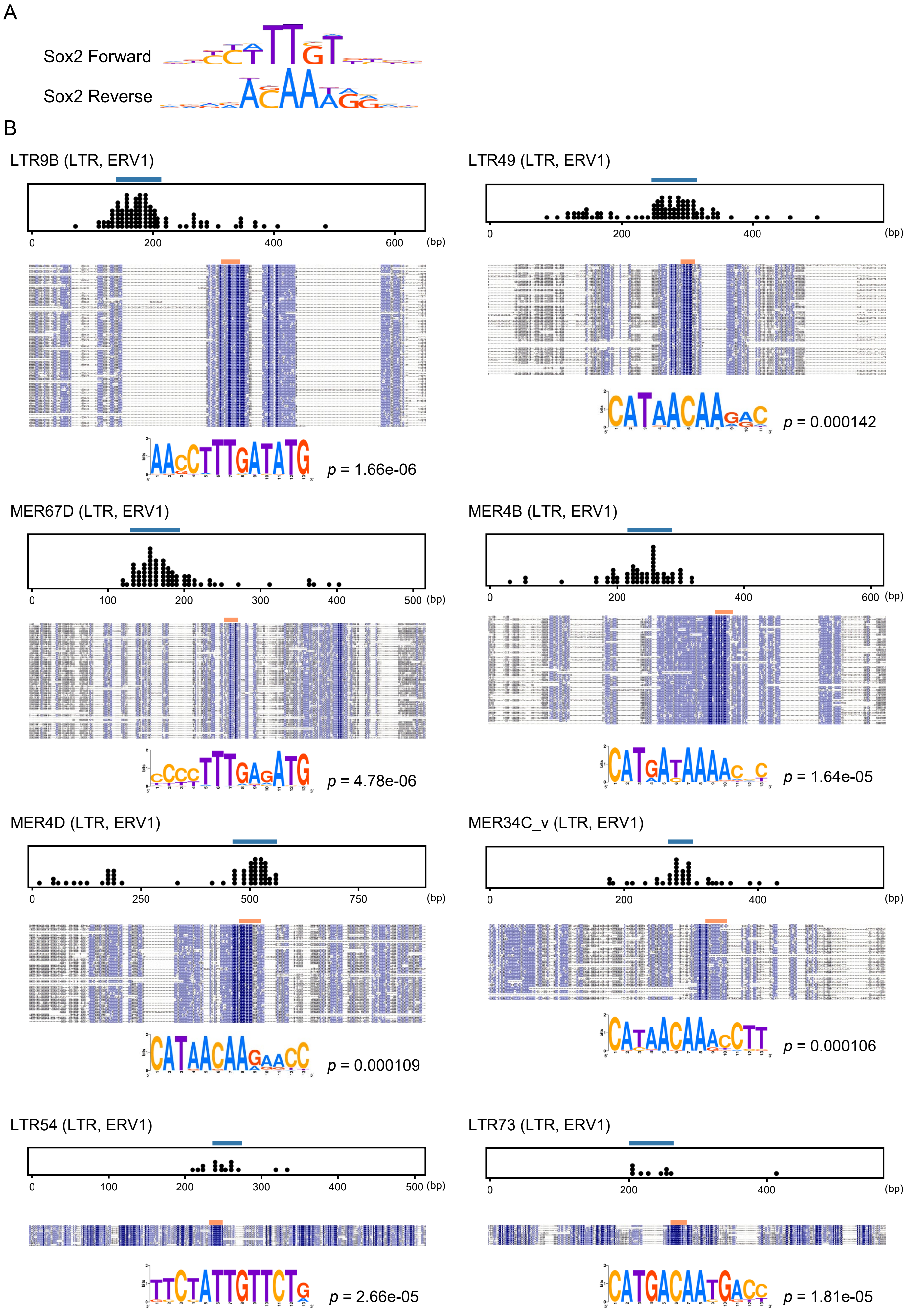
A
Sox2 Forward
Sox2 Reverse
B
LTR9B (LTR, ERV1)
0
200
400
600
(bp)
p = 1.66e-06
LTR49 (LTR, ERV1)
0
200
400
(bp)
p = 0.000142
MER67D (LTR, ERV1)
0
100
200
300
400
500
(bp)
p = 4.78e-06
MER4B (LTR, ERV1)
0
200
400
600
(bp)
p = 1.64e-05
MER4D (LTR, ERV1)
0
250
500
750
(bp)
p = 0.000109
MER34C_v (LTR, ERV1)
0
200
400
(bp)
p = 0.000106
LTR54 (LTR, ERV1)
0
100
200
300
400
500
(bp)
p = 2.66e-05
LTR73 (LTR, ERV1)
0
200
400
(bp)
p = 1.81e-05

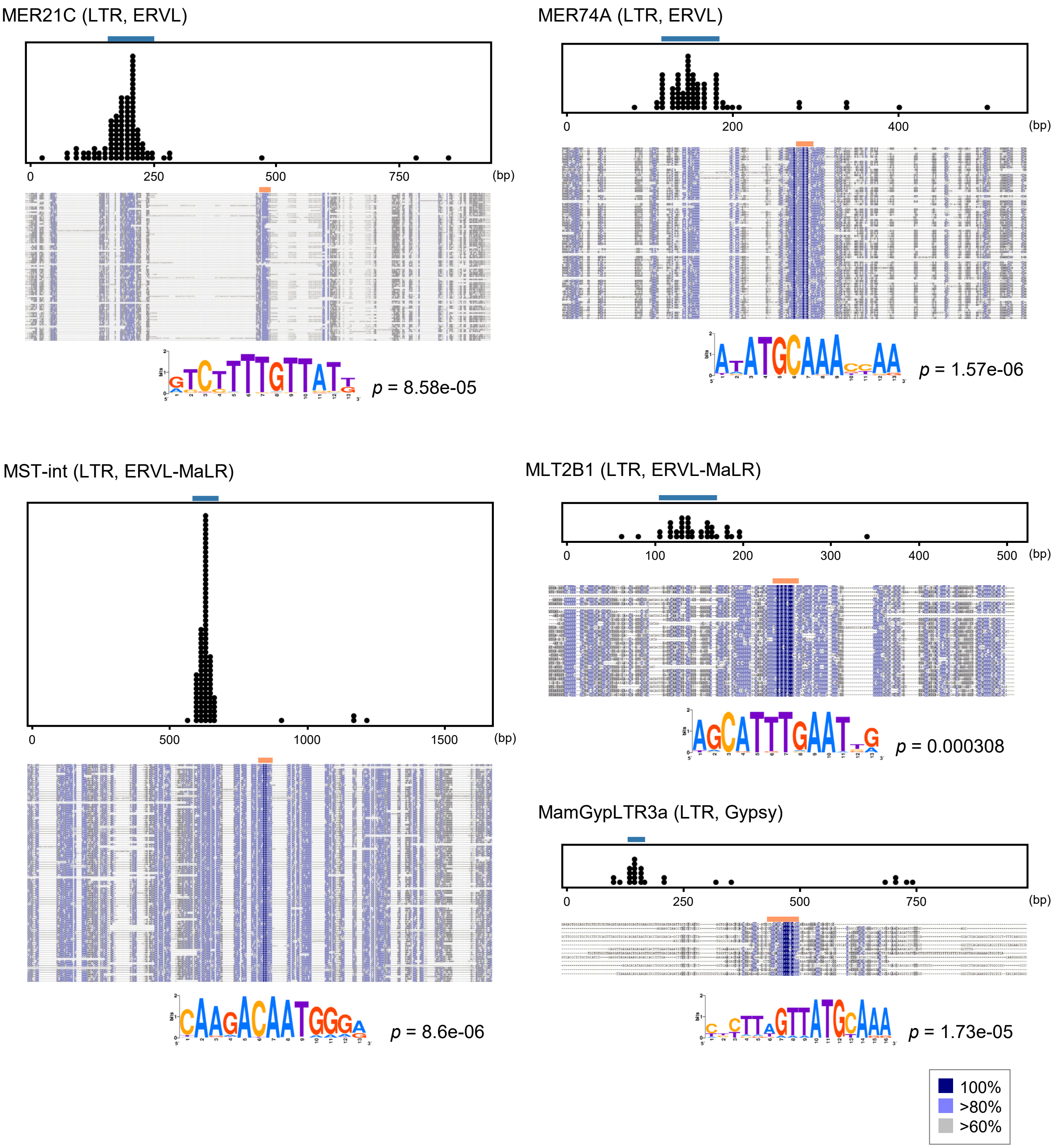

Fig. S6. Thirteen TE subfamilies contributing to the spread of Sox2-binding sites in ESCs. **A** The known Sox2-binding motif (SOX2_HUMAN.H11MO.0.A) obtained from the HOCOMOCO database. **B** Distribution of Sox2-binding sites along the consensus sequences of TEs that showed significant enrichment of binding sites in Fig. 3A. TE sequences with dense binding sites, marked by the blue line above the plots, are aligned. The orange lines above the alignments denote conserved Sox2-binding motifs, with the corresponding sequence logos shown below. *P*-values were calculated using FIMO analysis, comparing the known binding motif in (A) with the consensus sequences.

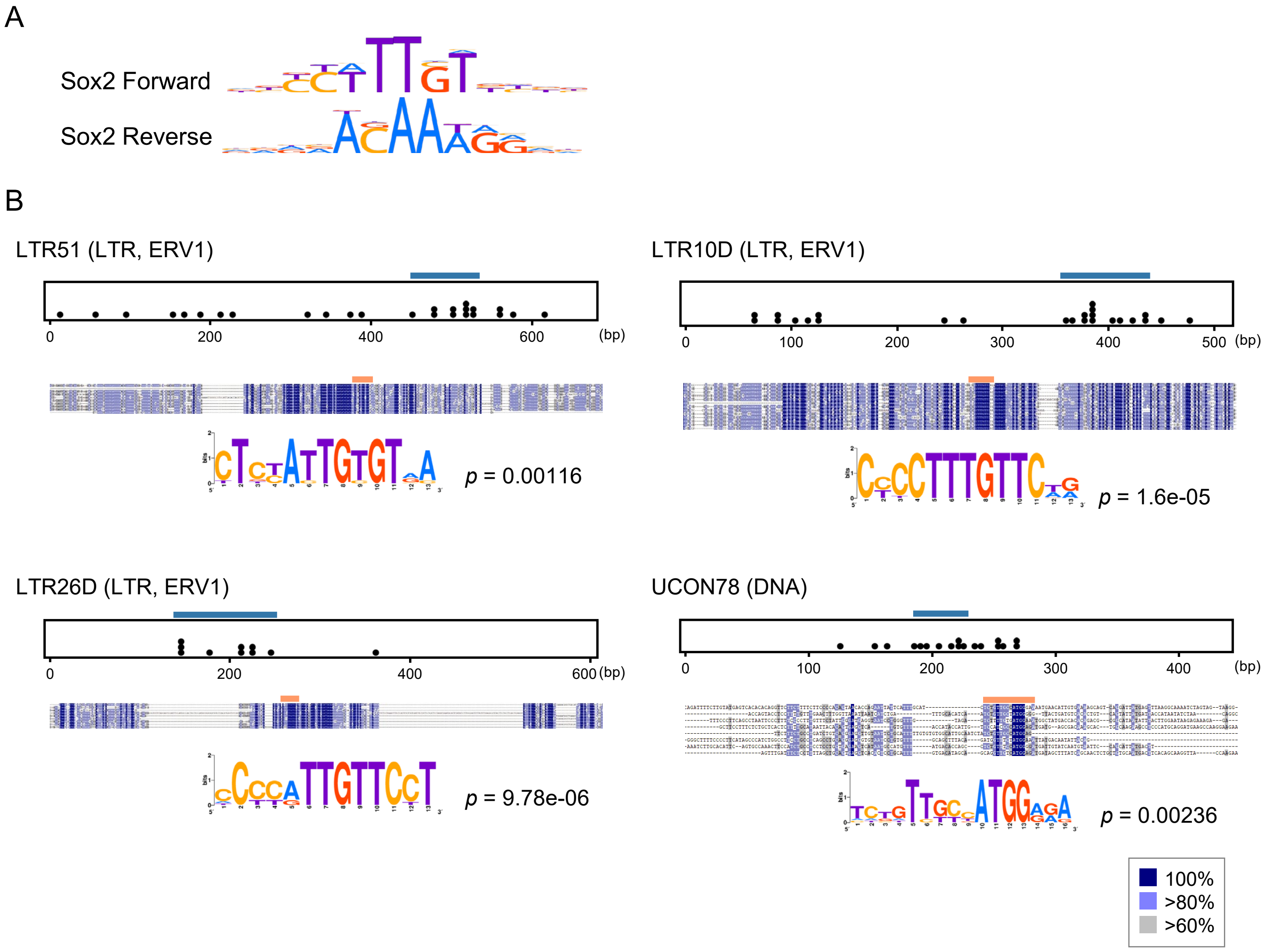

Fig. S7. Four TE subfamilies contributing to the spread of Sox2-binding sites in NPCs. **A** The known Sox2-binding motif (SOX2_HUMAN.H11MO.0.A) obtained from the HOCOMOCO database. **B** Distribution of Sox2-binding sites along the consensus sequences of TEs that showed significant enrichment of binding sites in Fig. 3B. TE sequences with dense binding sites, marked by the blue line above the plots, are aligned. The orange lines above the alignments denote conserved Sox2-binding motifs, with the corresponding sequence logos shown below. *P*-values were calculated using FIMO analysis, comparing the known binding motif in (A) with the consensus sequences.

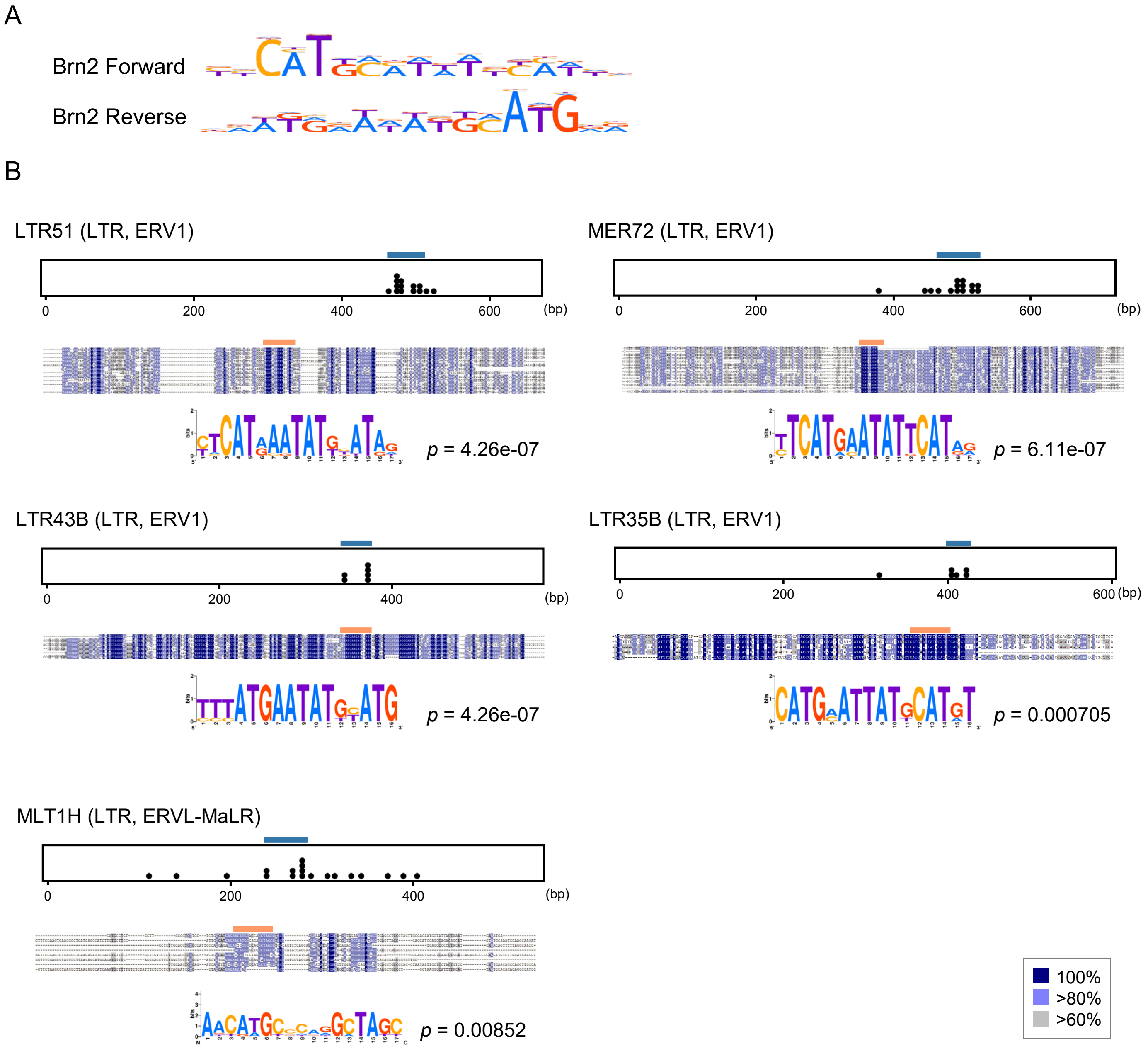

Fig. S8. Five TE subfamilies contributing to the spread of Brn2-binding sites in NPCs. **A** The known Brn2-binding motif (PO3F2_HUMAN.H11MO.0.A) obtained from the HOCOMOCO database. **B** Distribution of Brn2-binding sites along the consensus sequences of TEs that showed significant enrichment of binding sites in Fig. 3C. TE sequences with dense binding sites, marked by the blue line above the plots, are aligned. The orange lines above the alignments denote conserved Brn2-binding motifs, with the corresponding sequence logos shown below. *P*-values were calculated using FIMO analysis, comparing the known binding motif in (A) with the consensus sequences.

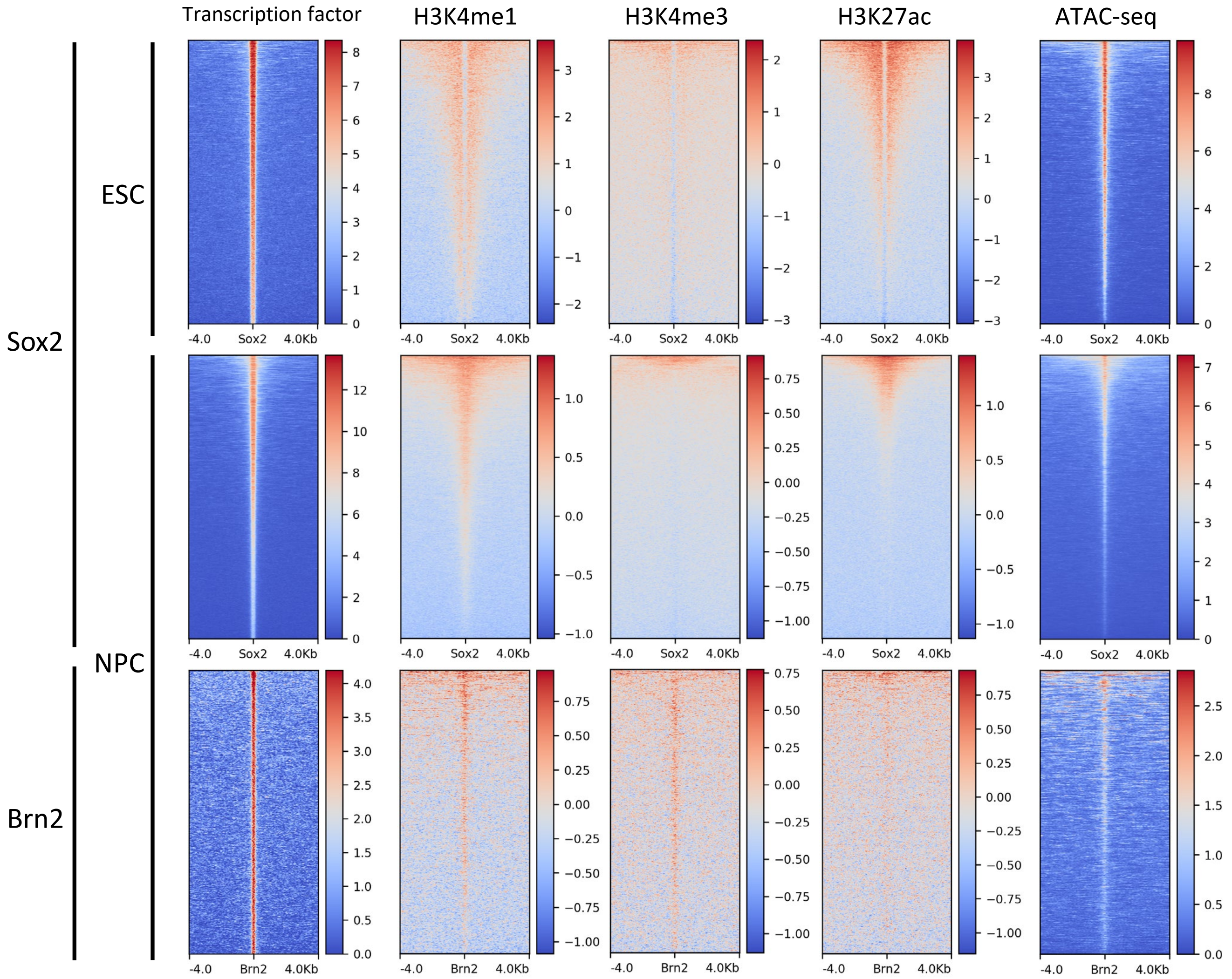

Fig. S9. Heatmaps of ChIP-seq and ATAC-seq signal intensities (RPKM normalization) centered on Sox2- and Brn2-binding sites within TEs. ChIP-seq signals for Sox2 and Brn2 transcription factors are shown on the left, and those for the three histone modifications (H3K4me1, H3K4me3, and H3K27ac) are shown in the center.

LTR9D (LTR, ERV1)

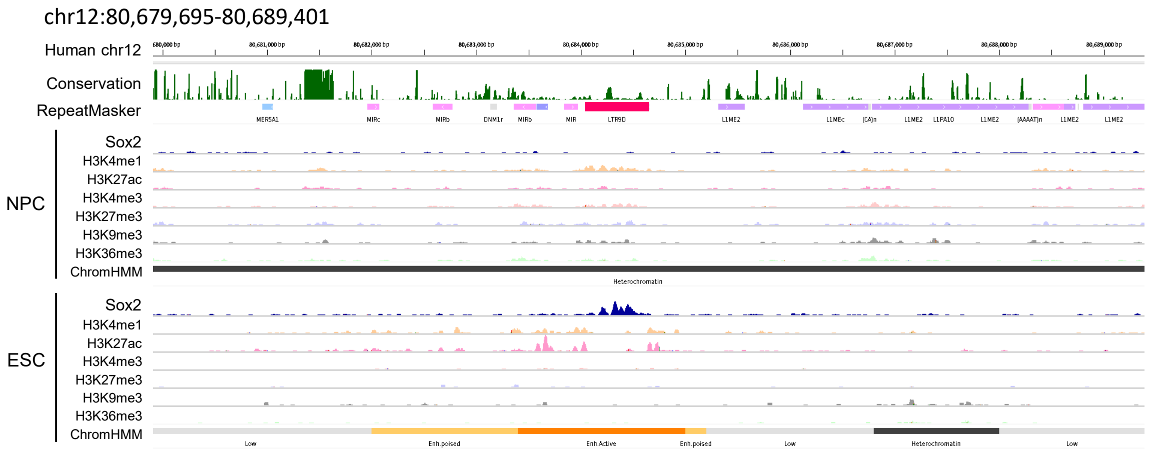

LTR49 (LTR, ERV1)

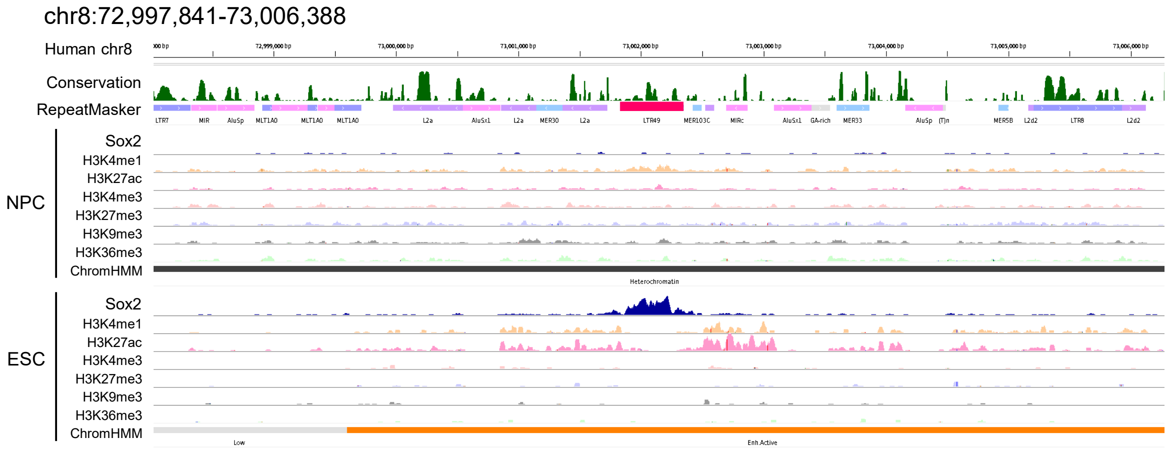

MER67D (LTR, ERV1)

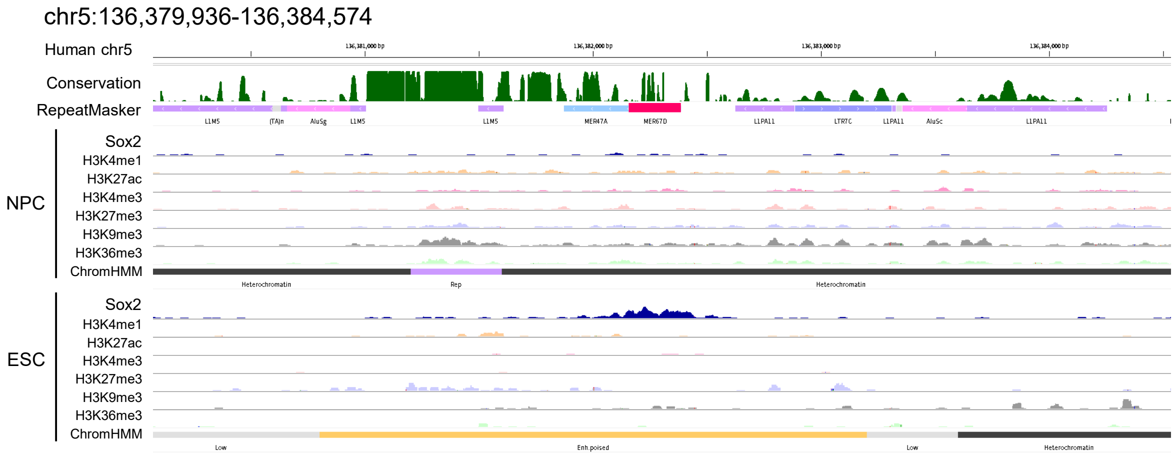

MER4B (LTR, ERV1)

chr9:25,392,023-25,402,601

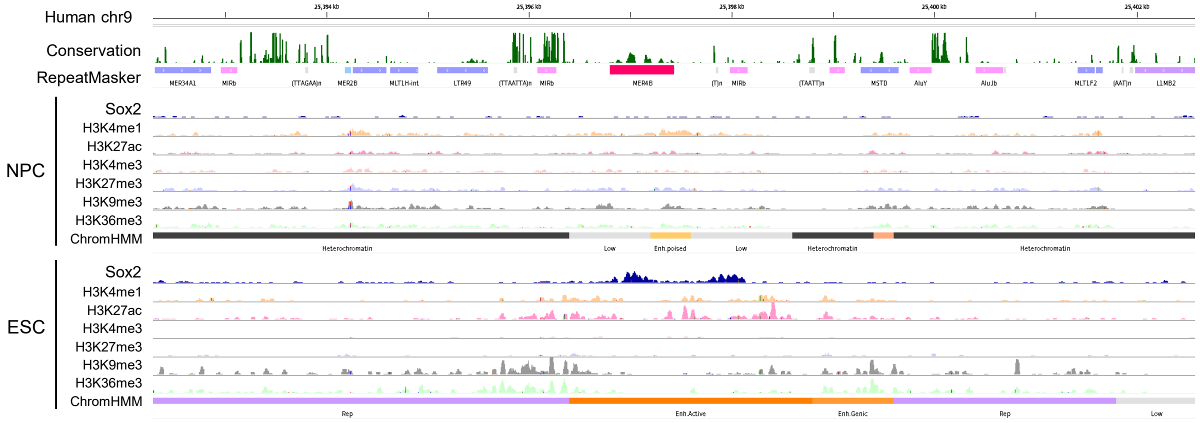

MER4D (LTR, ERV1)

chr18:54,066,851-54,072,895

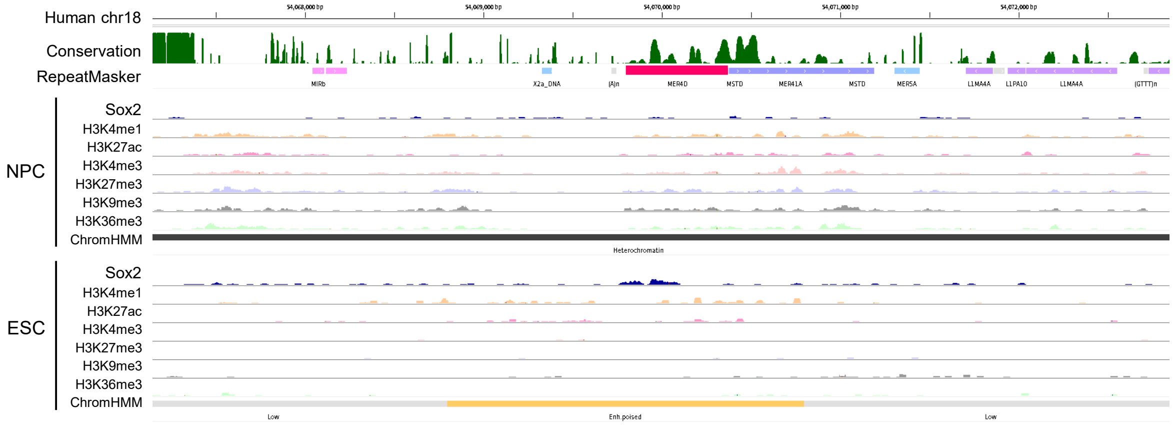

MER34C_v (LTR, ERV1)

chrX:16,038,699-16,047,825

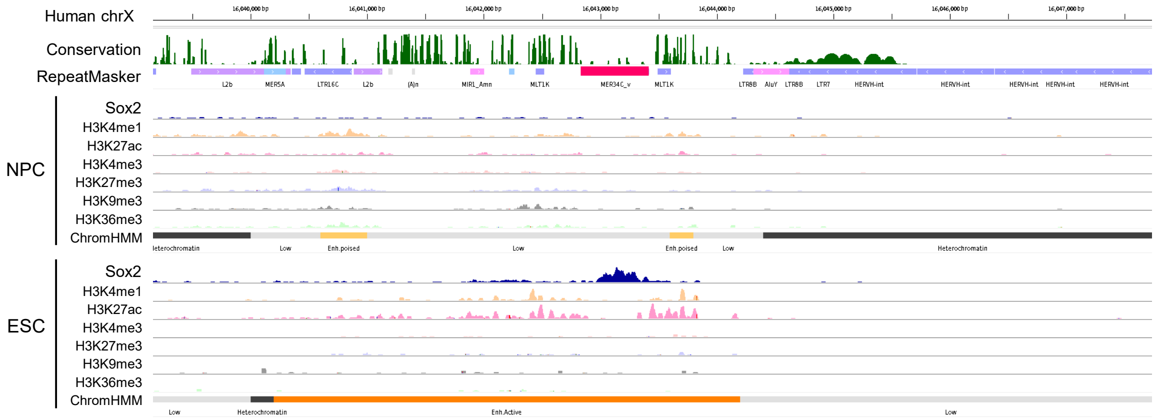

LTR54 (LTR, ERV1)

chr12:93,579,074-93,589,261

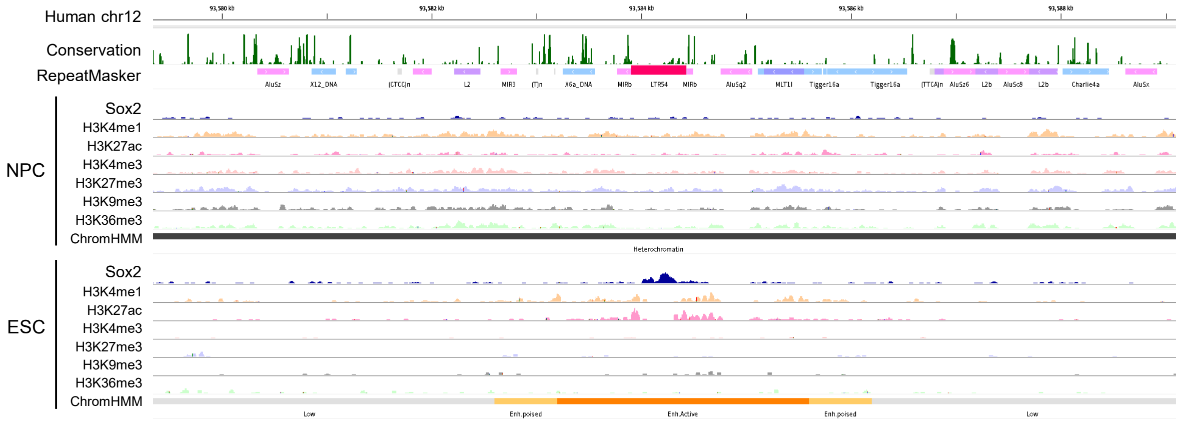

LTR73 (LTR, ERV1)

chr11:41,932,821-41,937,984

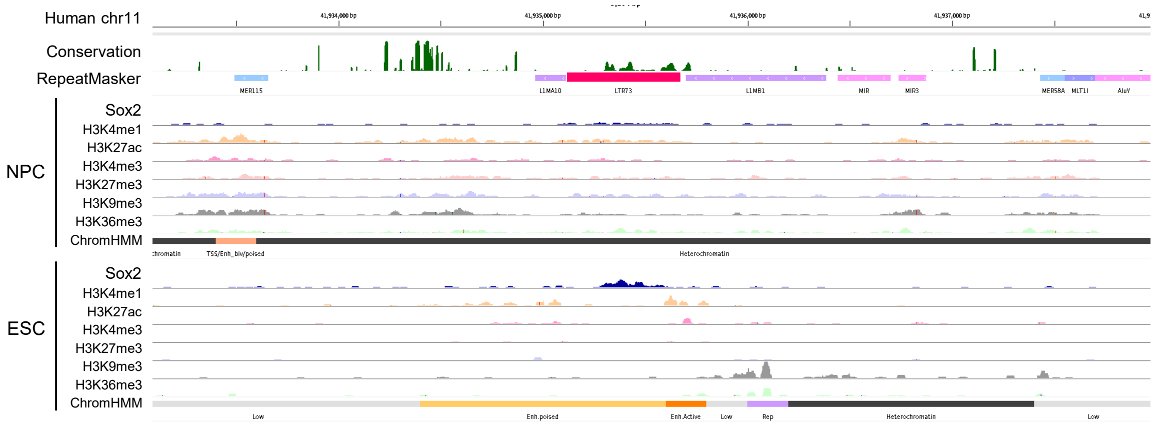

MER21C (LTR, ERVL)

chr19:33,030,427-33,034,708

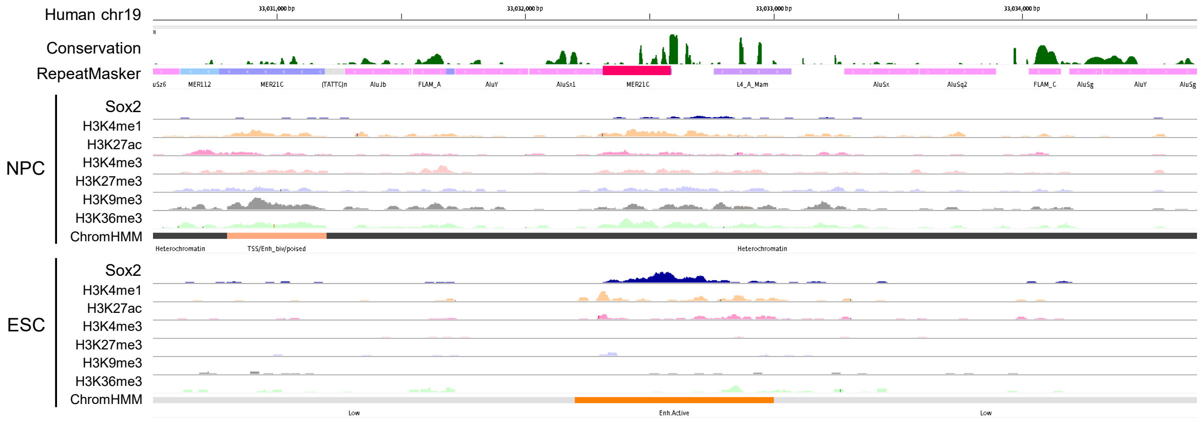

MER74A (LTR, ERVL)

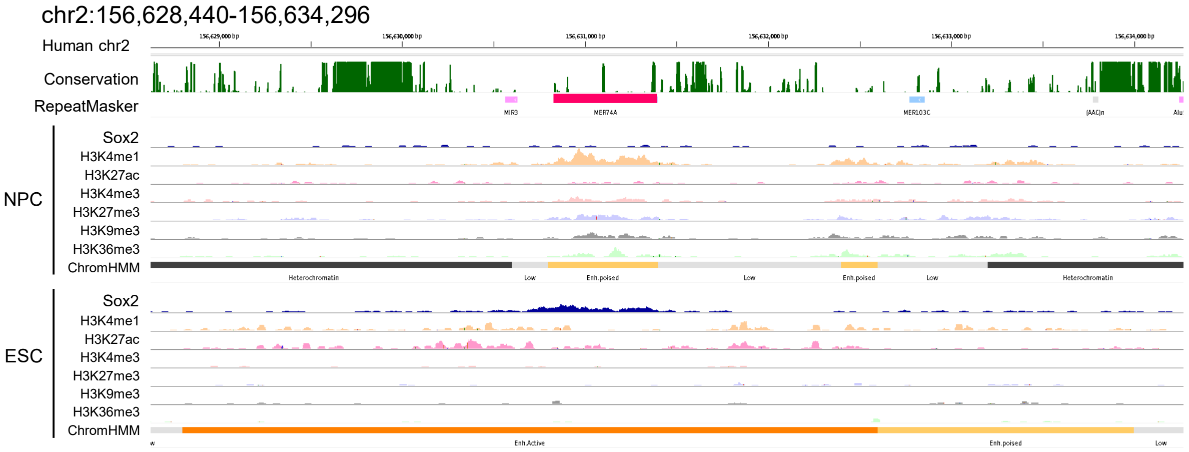

MST-int (LTR, ERVL-MaLR)

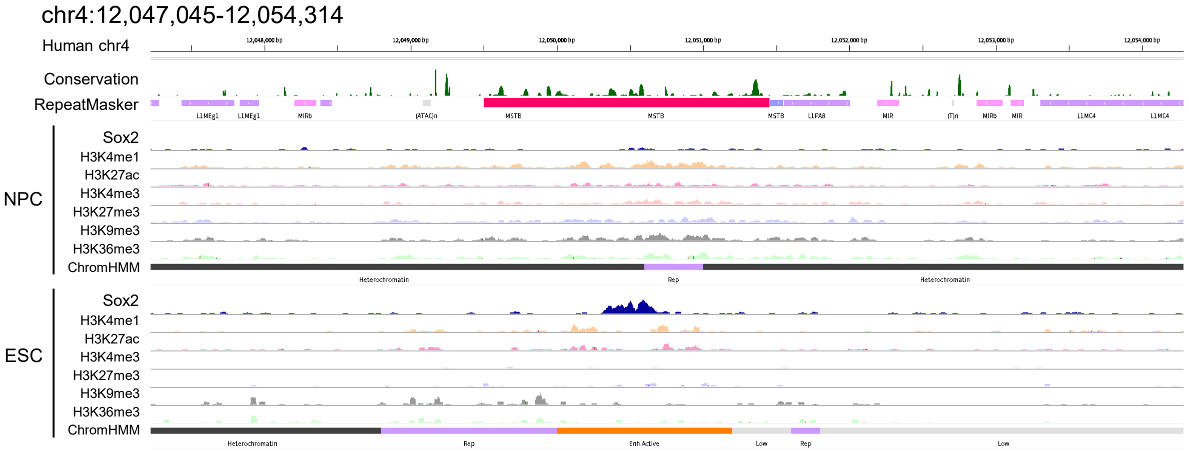

MamGypLTR3a (LTR, Gypsy)

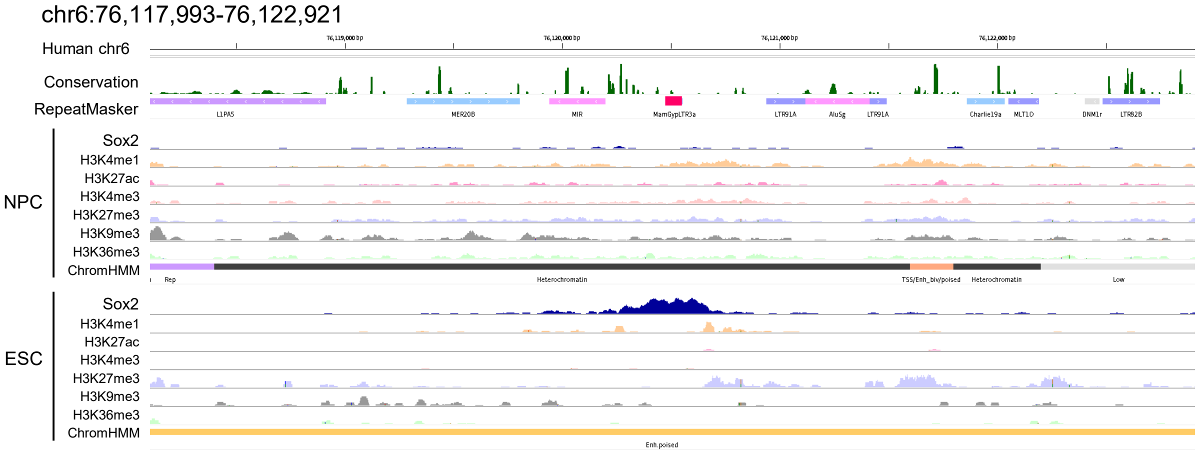

MLT2B1 (LTR, ERVL-MaLR)

chr1:87,721,136-87,725,793

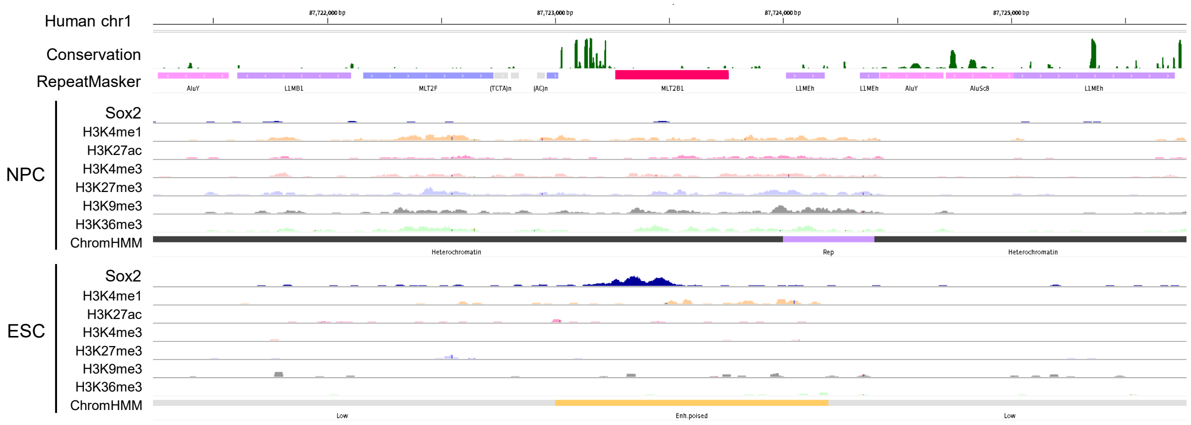

Fig. S10. Instances of TE loci bound by Sox2 in ESCs. The TE subfamilies and their genomic positions in the human genome assembly (hg38) are shown above the IGV browser panels. The top panel in each IGV browser view shows evolutionary conservation from phastCons30way (dark green) and TE annotations from RepeatMasker. The magenta bars in the RepeatMasker track indicate TEs where ChIP-seq peaks of Sox2 (blue) were detected in ESCs. In each panel, histone modifications and functional annotations from ChromHMM are shown in the middle and bottom tracks, respectively.

LTR51 (LTR, ERV1)

chr16:18,956,196-18,962,101

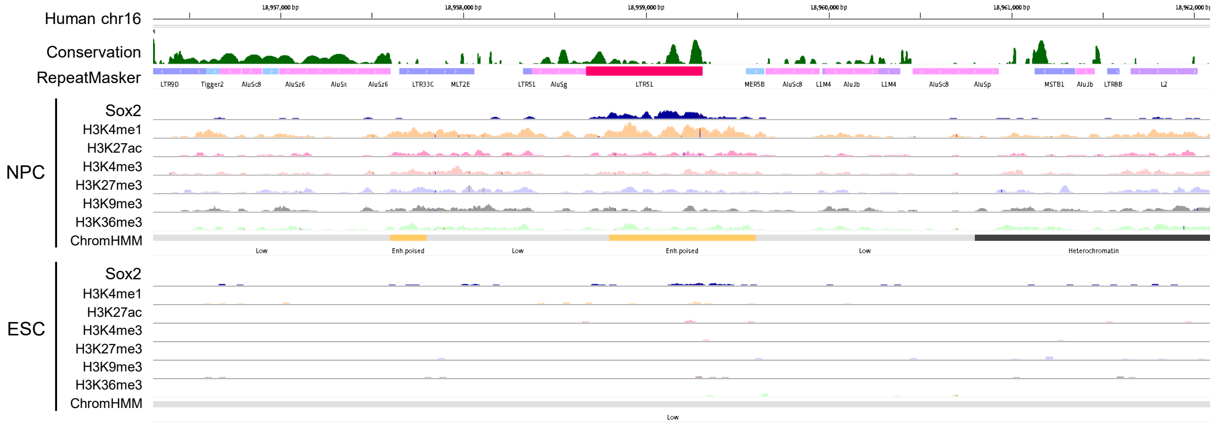

LTR10D (LTR, ERV1)

chr7:152,987,050-152,993,140

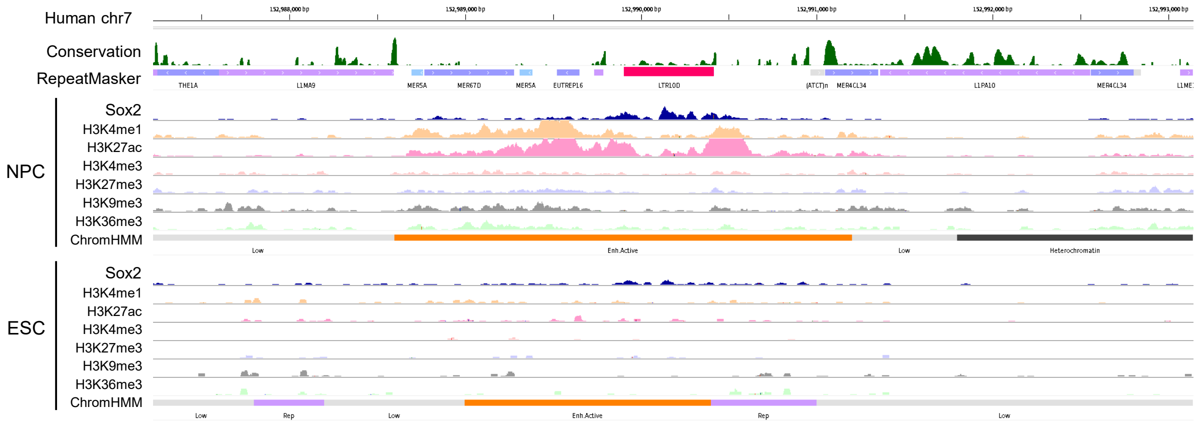

LTR26D (LTR, ERV1)

chr20:2,250,604-2,255,530

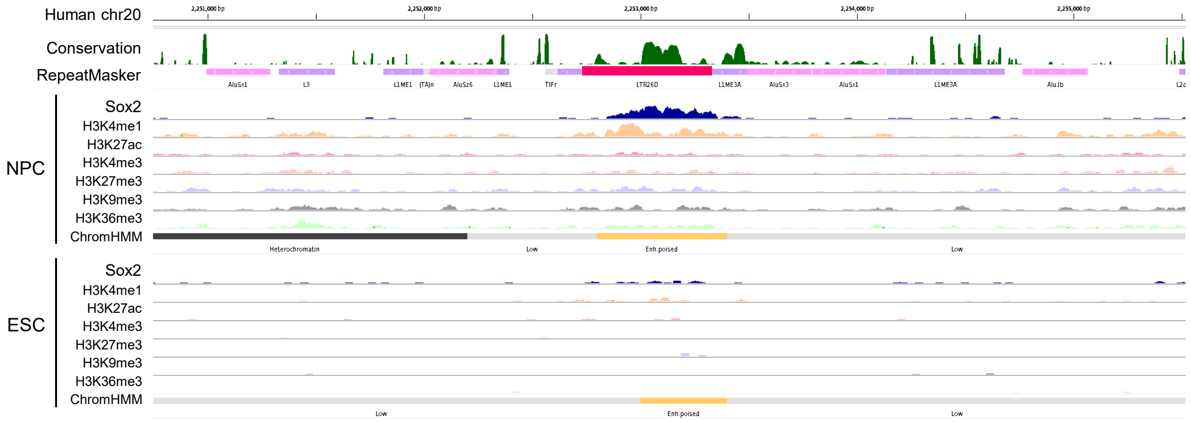

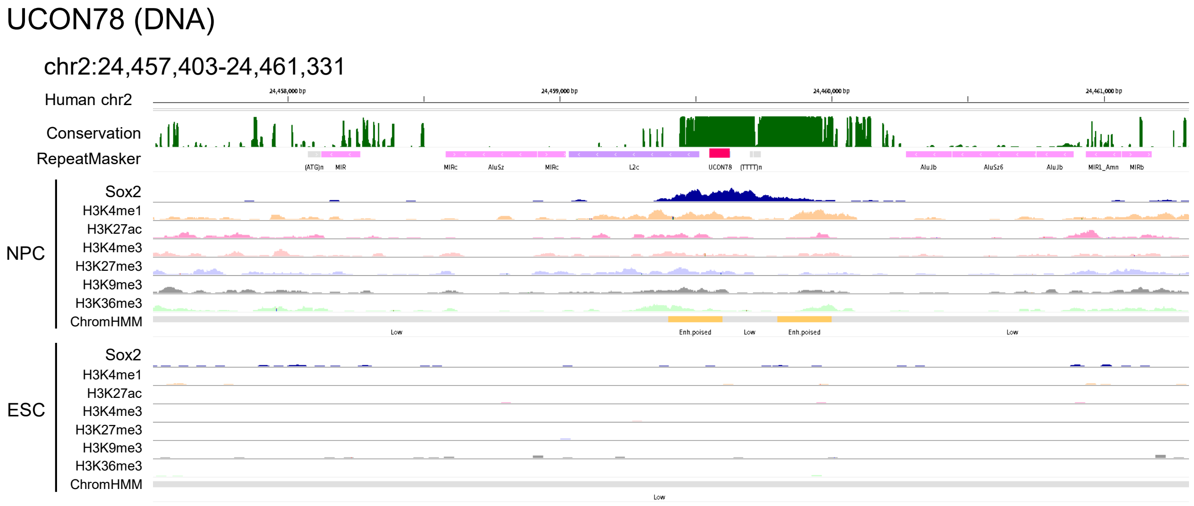

Fig. S11. Instances of TE loci bound by Sox2 in NPCs. The TE subfamilies and their genomic positions in the human genome assembly (hg38) are shown above the IGV browser panels. The top panel in each IGV browser view shows evolutionary conservation from phastCons30way (dark green) and TE annotations from RepeatMasker. The magenta bars in the RepeatMasker track indicate TEs where ChIP-seq peaks of Sox2 (blue) were detected in NPCs. In each panel, histone modifications and functional annotations from ChromHMM are shown in the middle and bottom tracks, respectively.

LTR51 (LTR, ERV1)

chr11:41,380,047-41,386,371

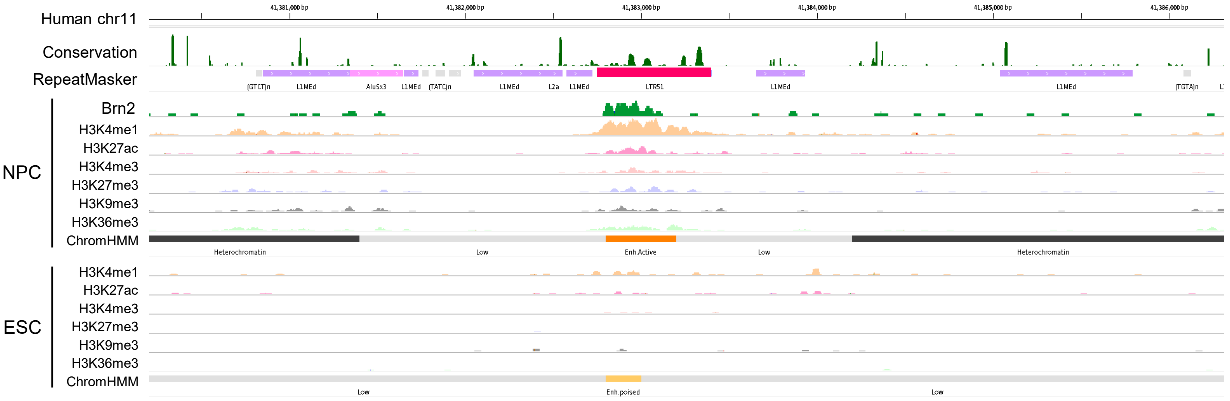

MER72 (LTR, ERV1)

chr1:100,783,482-100,788,595

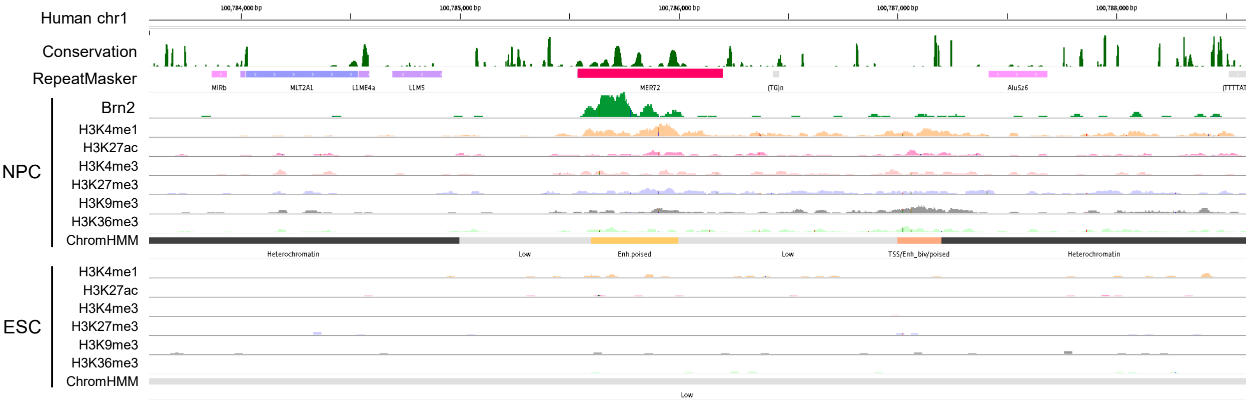

LTR43B (LTR, ERV1)

chr2:212,667,350-212,672,605

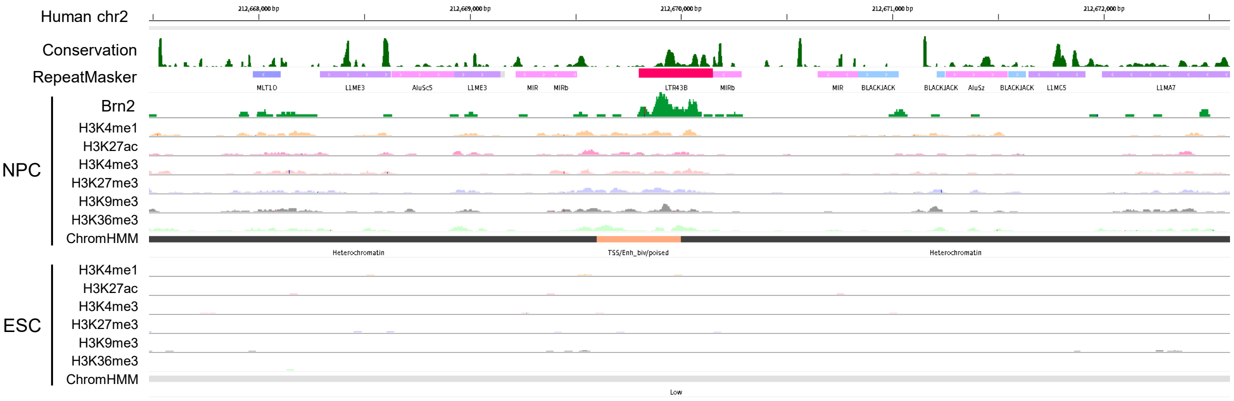

LTR35B (LTR, ERV1)

chr2:64,908,026-64,913,181

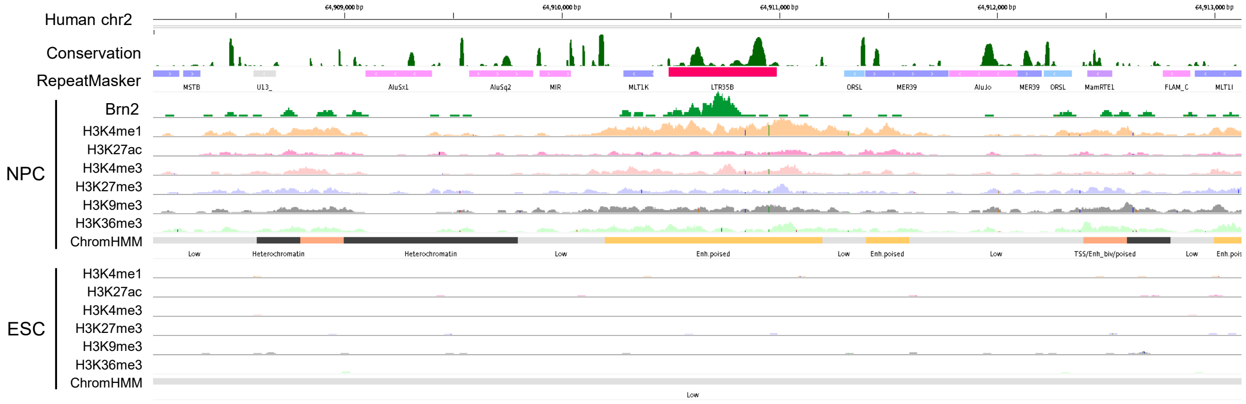

MLT1H (LTR, ERVL-MaLR)

chr14:46,155,181-46,162,362

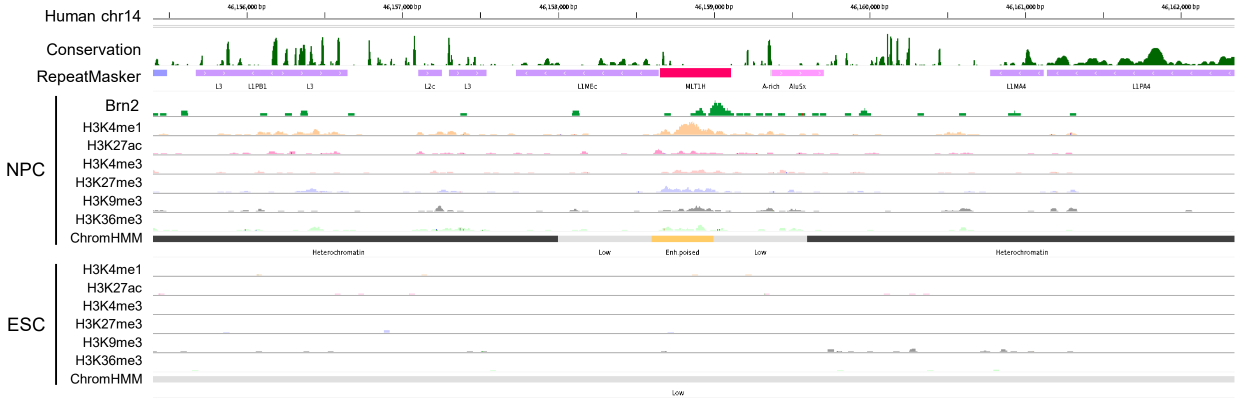

Fig. S12. Instances of TE loci bound by Brn2 in NPCs. The TE subfamilies and their genomic positions in the human genome assembly (hg38) are shown above the IGV browser panels. The top panel in each IGV browser view shows evolutionary conservation from phastCons30way (dark green) and TE annotations from RepeatMasker. The magenta bars in the RepeatMasker track indicate TEs where ChIP-seq peaks of Brn2 (green) were detected in NPCs. In each panel, histone modifications and functional annotations from ChromHMM are shown in the middle and bottom tracks, respectively.

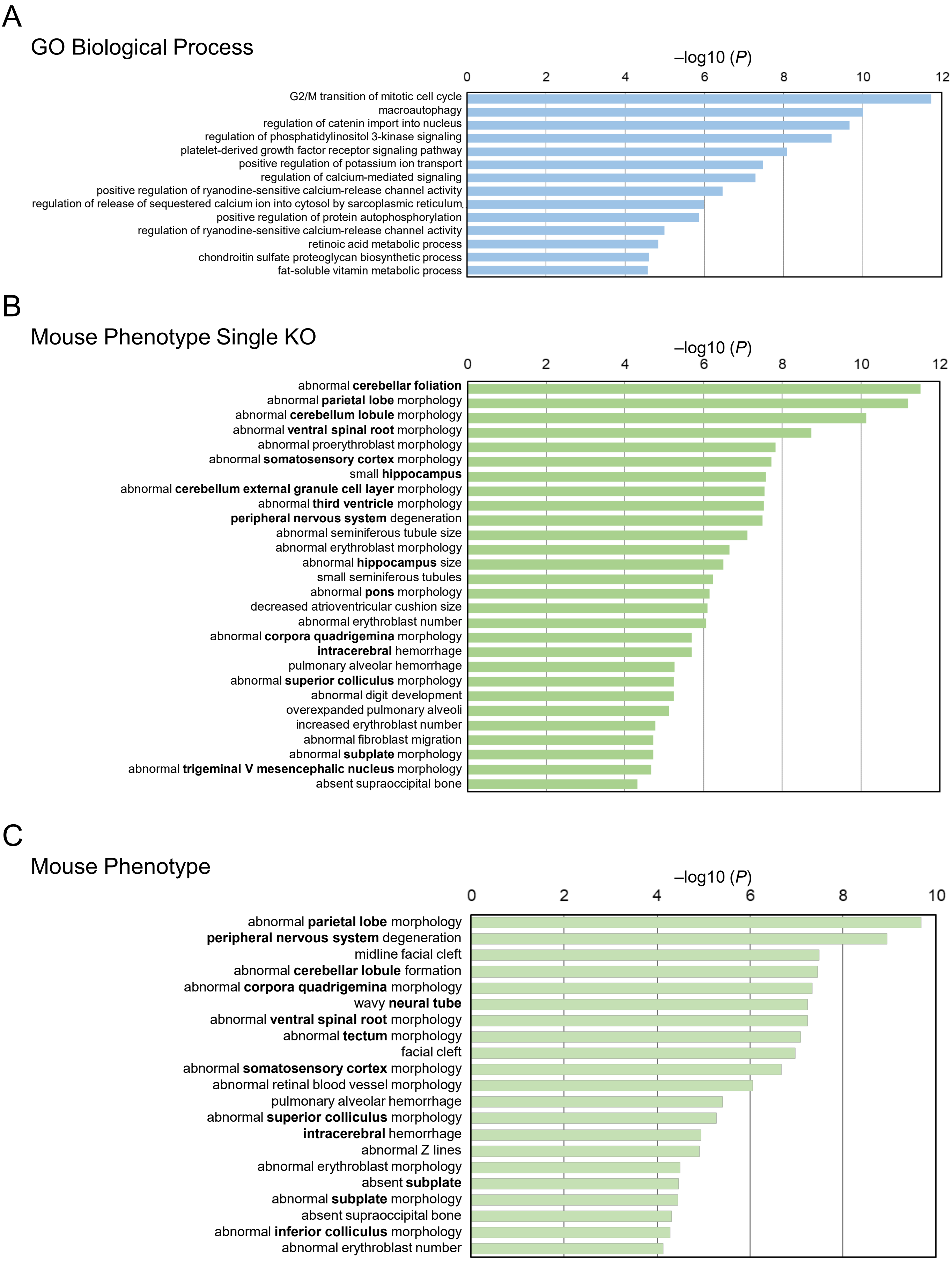

Fig. S13. A–C GREAT gene ontology analysis of 3,685 NPC-specific Sox2-binding TEs whose nearest genes are upregulated in NPCs (Fig. 7C). The –log10 (*P*-value) is shown for the GO Biological Process (**A**), Mouse Phenotype Single KO (**B**), and Mouse Phenotype (**C**) categories in the GREAT analysis. GO terms related to neurogenesis are highlighted in bold.

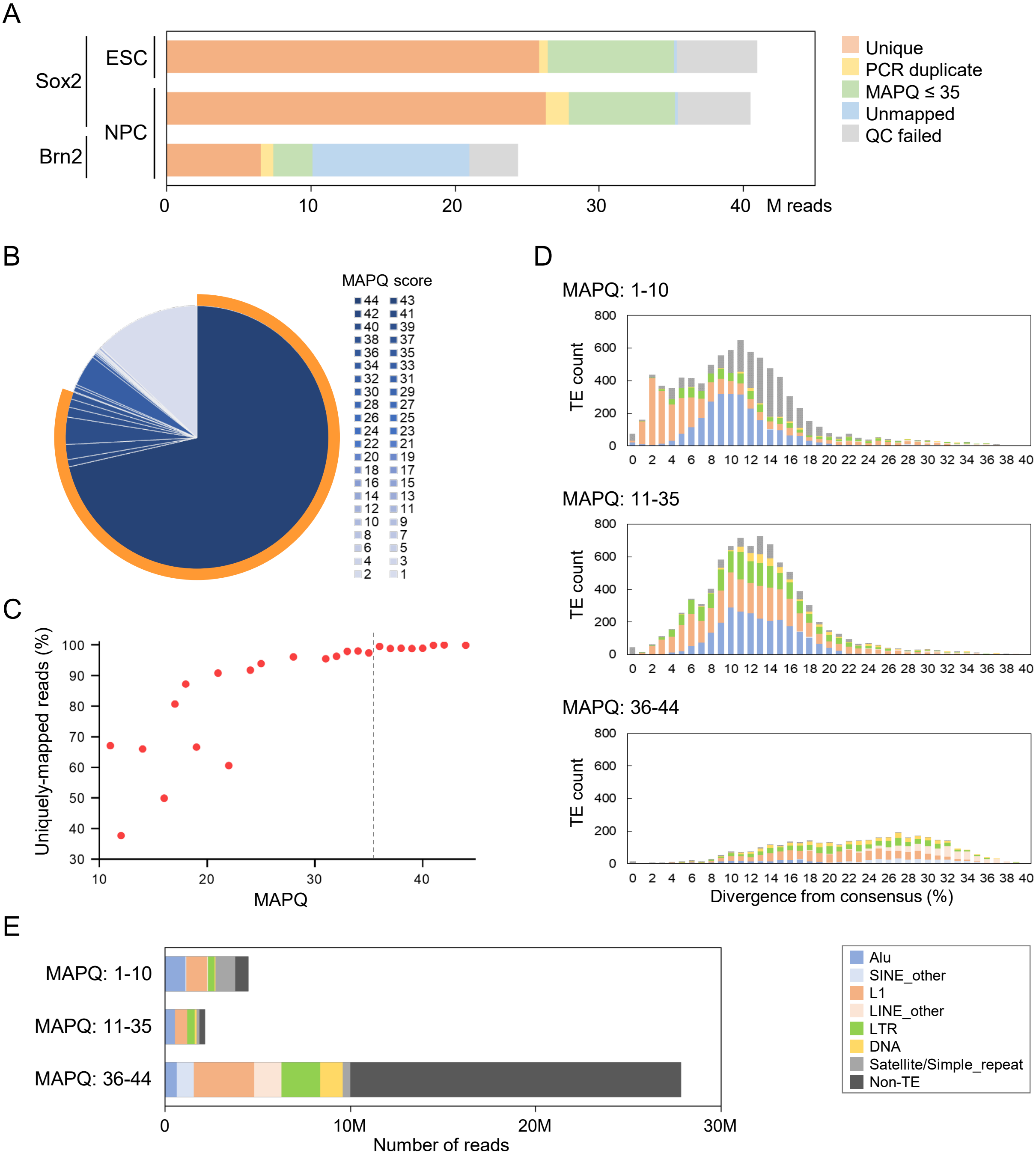

Fig. S14. Quality control of ChIP-seq reads used in this study. **A** Number of Sox2 ChIP-seq reads in ESCs and NPCs. Uniquely mapped reads used for peak calling were obtained by aligning quality-checked (QC-passed) reads to the human genome assembly (hg38), followed by removal of low-MAPQ reads and PCR duplicates. **B** Distribution of MAPQ scores among 34.5 million mapped Sox2 ChIP-seq reads from NPCs. Reads with MAPQ > 35 (indicated by the orange line; 80.7%) were used for subsequent analyses. **C** Proportion of uniquely mapped reads within each MAPQ score category, assessed using blastn searches of 1,000 randomly selected reads per category. The dotted line indicates the MAPQ threshold adopted in this study. **D** Age distribution of TEs among 10,000 randomly selected mapped reads from each MAPQ category, showing a higher proportion of younger TEs (with lower sequence divergence) at lower MAPQ scores. **E** Number of TE-derived and non-TE–derived mapped reads across the three MAPQ categories, estimated from 10,000 randomly selected reads shown in (D).

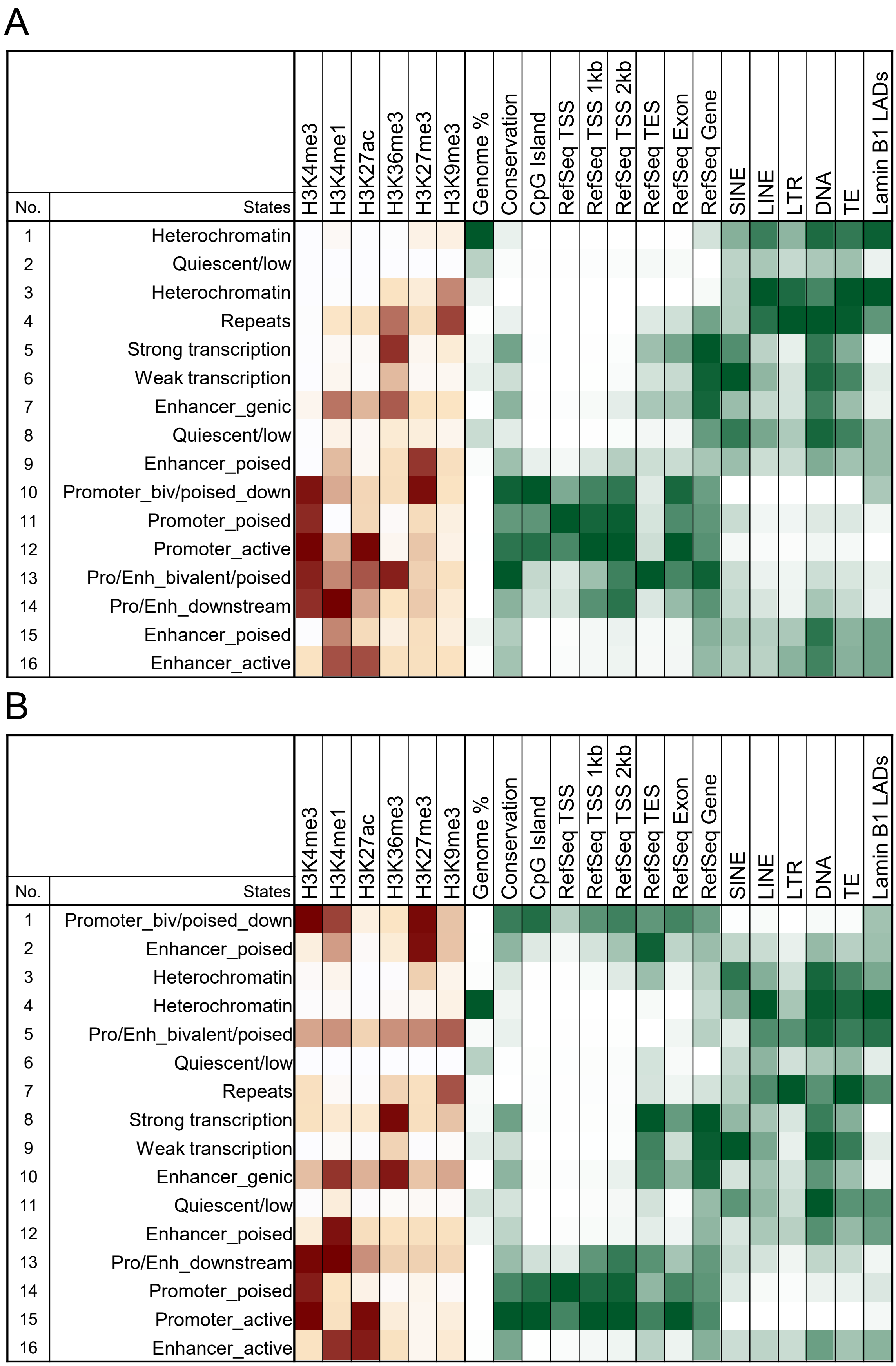

Fig. S15. A–B The 16 functional categories classified by ChromHMM based on the epigenetic states of ESC (**A**) and NPC (**B**) as well as their genomic states. Heatmap colors represent the proportions of the histone modification signals (red) and those of the genomic feature (green). Pro, promoter; Enh, enhancer; biv, bivalent; down, downstream; TSS, transcription start site; TES, transcription end site.
